# Triadic percolation induces dynamical topological patterns in higher-order networks

**DOI:** 10.1093/pnasnexus/pgae270

**Published:** 2024-07-09

**Authors:** Ana P Millán, Hanlin Sun, Joaquín J Torres, Ginestra Bianconi

**Affiliations:** Electromagnetism and Matter Physics Department, Institute “Carlos I” for Theoretical and Computational Physics, University of Granada, Granada E-18071, Spain; Nordita, KTH Royal Institute of Technology and Stockholm University, Stockholm SE-106 91, Sweden; Electromagnetism and Matter Physics Department, Institute “Carlos I” for Theoretical and Computational Physics, University of Granada, Granada E-18071, Spain; Centre for Complex Systems, School of Mathematical Sciences, Queen Mary University of London, London E1 4NS, UK; The Alan Turing Institute, London NW1 2DB, UK

**Keywords:** percolation, higher-order interactions, spatial networks, nonlinear dynamics, TDA

## Abstract

Triadic interactions are higher-order interactions which occur when a set of nodes affects the interaction between two other nodes. Examples of triadic interactions are present in the brain when glia modulate the synaptic signals among neuron pairs or when interneuron axo-axonic synapses enable presynaptic inhibition and facilitation, and in ecosystems when one or more species can affect the interaction among two other species. On random graphs, triadic percolation has been recently shown to turn percolation into a fully fledged dynamical process in which the size of the giant component undergoes a route to chaos. However, in many real cases, triadic interactions are local and occur on spatially embedded networks. Here, we show that triadic interactions in spatial networks induce a very complex spatio-temporal modulation of the giant component which gives rise to triadic percolation patterns with significantly different topology. We classify the observed patterns (stripes, octopus, and small clusters) with topological data analysis and we assess their information content (entropy and complexity). Moreover, we illustrate the multistability of the dynamics of the triadic percolation patterns, and we provide a comprehensive phase diagram of the model. These results open new perspectives in percolation as they demonstrate that in presence of spatial triadic interactions, the giant component can acquire a time-varying topology. Hence, this work provides a theoretical framework that can be applied to model realistic scenarios in which the giant component is time dependent as in neuroscience.

**Significance Statement**

In the brain, there are many scenarios in which neural activity displays spatio-temporal patterns leading to a nonstationary dynamics of the connectivity of the functional networks. Yet, the standard theory of percolation, highly successful in describing how the network connectivity responds to damage, falls short in explaining these intricate phenomena. Our research bridges this gap by introducing spatial triadic percolation highlighting the importance of considering percolation in presence of higher-order triadic interactions. We demonstrate that triadic percolation induces dynamical topological patterns of the giant component on spatial networks. This result opens the way to understand the fundamental mechanisms for the emergence of a time-varying topology of the connected components not only in neuroscience but also in complex systems more broadly.

Percolation (1–7) is a pivotal critical phenomenon that captures the nonlinear response of a network to the damage of its links. Thus, percolation has a wide range of applications in complex systems including brain and infrastructure networks (8–11). The dynamical model usually implied by the percolation processes describes cascades or avalanches of failure events, typically reaching a static absorbing state, which might result in a dismantled network. Hence, the traditional approach to percolation fails to describe the situation encountered for instance in neuroscience where their giant component of functional brain networks dynamically varies in time without reaching a static configuration. In order to capture this scenario, higher-order triadic interactions have recently been shown to be a key, leading to the formulation of triadic percolation (12).

Higher-order networks (13–18) include interactions between two or more nodes. These generalized network structures are attracting large interest because they are transforming significantly our understanding of the interplay between topology and dynamics of complex networks. Indeed the structure of higher-order networks captures the underlying topology and geometry of the data, with applications from ecology (19, 20) to cancer research (21–23). Evidence of higher-order coupling appears in several natural systems (13–18), including brain dynamics, chemical interaction networks, and the climate (24–31). Higher-order interactions have the potential to dramatically change the emergent behavior of a dynamical model (13, 18), as evidenced in percolation (12, 32–34), synchronization (35–38), diffusion (39, 40), game theory (41), and contagion and consensus dynamics (42–46).

Triadic interactions (12, 19, 47–49) are higher-order interactions that occur when one or more nodes regulate the interaction between two other nodes. This is the role for instance of glia cells in neuronal networks, as they modulate synaptic interactions between neuron pairs (50). In the brain, presynaptic inhibition and facilitation are further examples of triadic interactions. Indeed, axo-axonic synapses involve interneurons that induce presynaptic inhibition or facilitation, which reduce or enhance, respectively, postsynaptic response (51, 52). Similarly, in ecological systems, the presence of a third species may modulate the interaction between two given species (19, 47).

Recently, in Ref. (12), it was shown that triadic percolation, in which triadic interactions up- or down-regulate links, defines a fully fledged dynamical process in which the giant component becomes dynamical, and its size undergoes a period-doubling and a route to chaos. However, Ref. (12) only addresses triadic percolation on a random graph while in a large variety of cases, real-world networks have a geometric embedding, such is the case for instance for neuronal networks (53, 54), communication and transportation systems (55). For many of these systems, an exponential wiring cost fits the observed spatial distribution of link lengths (10, 56–65). Despite many advances in the study of percolation on spatial random networks and lattices (6, 7, 10, 11, 66–69), their analysis in the presence of a geometric embedding remains an important theoretical challenge.

In this work, we address the study of triadic percolation defined on spatially embedded networks with triadic interactions. We show that, since in these networks interactions are local, triadic percolation induces a spatial structure on the giant component, leading to triadic percolation patterns, which display a distinct time-varying geometry and topology. Here, we investigate the spatio-temporal complexity of the observed dynamics of the triadic percolation patterns, which goes beyond the basic statistics and dynamics of the size of the giant component. To explore these findings, we investigate the spatial properties of the triadic percolation patterns with topological data analysis (TDA) (70–73), and information theory tools (74–76), identifying the emergence of three distinct types of patterns: small clusters, stripes, and octopus (or complex) patterns. We launch an in-depth numerical investigation of the dynamics of these patterns: we show evidence of intermittency with patterns of different types occurring in the same time-series, and we provide evidence of periodic (blinking) spatio-temporal behavior of the patterns. These analyses allow us to reveal the topological and dynamical nature of the phase diagram of triadic percolation in spatial networks.

Triadic percolation on spatial networks captures basic mechanisms at play in a large variety of spatially embedded systems with triadic interactions, notably including neuronal and brain networks. In these biological systems, active nodes can be associated with neurons having large firing rates which can induce in some cases synaptic facilitation and axo-axonic inhibition in other situations. The complex interplay among these different synapse regulatory processes could be in the origin of the neural activity patterns observed in different brain areas including, for instance, the reported patterns during mammalian neocortex development (77), and visual cortex (78) and hippocampus (79) neural activity patterns.

In order to reveal the basic implications of having spatially embedded triadic interactions, here we keep our model very general. At the same time, the stylized nature of this model proposes a framework that combines percolation theory, topology and nonlinear dynamics that can become the starting point for more realistic models of specific scenarios in which the giant component is strongly time-dependent. Thus, the observed phenomena open new perspectives for the understanding of brain and neuronal networks function.

## Triadic percolation on spatial networks with triadic interactions

**Fig. 1.**
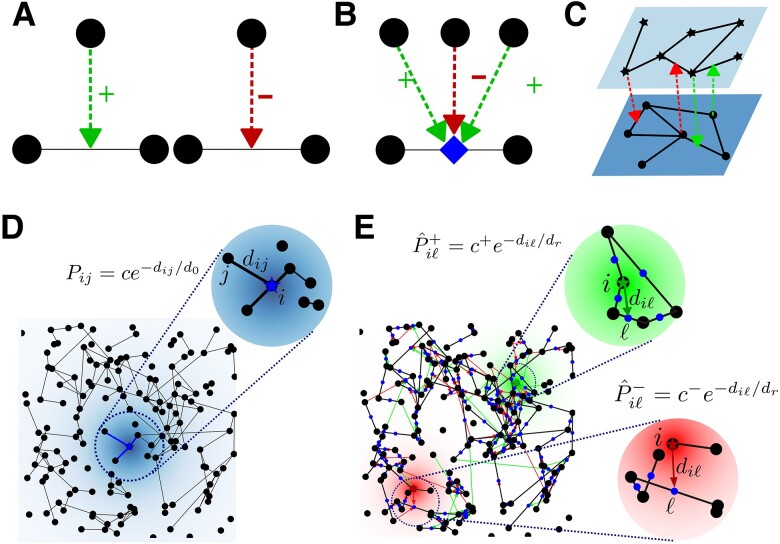
Network and triadic percolation models. A) Illustration of the triadic interaction in which one node regulates the link between two other nodes with a positive (left image) or negative (right image) effect. B) Triadic interactions allow for several regulator nodes for each link. In this case, the presence of a link is determined by a function taking into account both positive and negative regulatory interactions. C) Triadic two-layer network, where each layer regulates the interactions in the other layer. D) Illustration of the spatial structural network. We highlight the local connectivity mechanism in the zoomed area. Two nodes *i* and *j* are connected by a structural link with a probability $P_{ij}$ that depends on the Euclidean distance $d_{ij}$ between the two nodes, on the typical length for structural links $d_{0}$, and on the parameter *c* that determines the average degree of the structural network. The probability is schematically indicated by the shaded blue color in the top zoomed area. E) Illustration of the spatial network with triadic interactions. The higher-order network is formed by the spatial structural network shown in D) and the triadic interactions. The higher-order network with triadic interactions is formed by nodes (black circles), structural links (black lines the center of each structural link, indicating its location, is shown by smaller blue circles), and triadic regulatory interactions which can either be positive (green arrows) or negative (red arrows) as depicted in the zoomed areas. A positive regulatory interaction between a node *i* and a structural link ℓ is added with a probability ${\hat{P}}_{i\ell }^{+}$, schematically indicated by the shaded green color, while a negative regulatory interaction is added with probability ${\hat{P}}_{i\ell }^{-}$, schematically indicated by a shaded red color. The probabilities ${\hat{P}}_{i\ell }^{+}$ and ${\hat{P}}_{i\ell }^{-}$ depend on the Euclidean distance $d_{il}$ between a node and the link it regulates, on the typical range $d_{r}$ for regulatory links, and on the parameters $c^{+}$ and $c^{-}$ determining the average degree of positive and negative regulatory interactions respectively.

Triadic interactions are higher-order interactions that occur when one node regulates the interaction between two other nodes, either positively or negatively (see Fig. 1A, B). Positive regulations facilitate the interaction between the two nodes while negative regulations inhibit their interaction. Interestingly, triadic interactions can also exist in a multilayer setting (80) when the regulator nodes are of a different type (see Fig. 1C). A typical example of this latter type of triadic interaction is present in neuronal systems, where glia cells, for example, can either facilitate or inhibit the synaptic signals between neurons. Another example is the existence of interneurons that establish axo-axonic synapses that can induce presynaptic facilitation and inhibition of other synapses. For the sake of simplicity, in this work we consider the simple scenario in which the regulator nodes belong to the same structural network whose links are regulated by triadic interactions, as it occurs in presynaptic inhibition and facilitation. However, the multilayer scenarios can be a natural extension to the proposed framework.

We consider a higher-order spatial network with triadic interactions embedded in a 2D torus (square of size *L* with periodic boundary conditions). This higher-order network can be thought of as a multilayer network (80) formed by a structural network $G_{s}$ and a regulatory network $G_{r}$. The structural network $G_{s}=(V,E)$ comprises the set *V* of nodes and the set *E* of structural links between them. We indicate with *N* the number of nodes in *V*, i.e. N=|V|. The regulatory network $G_{r}=(V,E,W),$ on the other hand, is a signed factor network (bipartite network) formed by the nodes in *V* (called regulators), the structural links in *E* (playing the role of factor nodes), and the signed regulatory interactions between them, specified in the set *W*. Note that the signs of regulatory interactions are considered here as a property of the regulatory link rather than the regulator node. This means that a given regulator node can positively regulate some links (thus called positive regulator to these links) and negatively regulate some other links (thus called negative regulator to these links).

The spatial structural network is constructed according to the Waxman model (81) (see Fig. 1). This model accounts for local interactions as links between pairs of nodes are drawn with a probability that decays exponentially with their distance as found in several brain structural investigations (56, 57). Similarly, our model of spatial triadic interactions also establishes regulatory interactions among pairs of nodes and structural links with a probability that decays exponentially with their distance (see Materials and methods for details of the model). The model depends on few parameters: $d_{0}$ and $d_{r}$ indicate the typical range of interactions for structural and regulatory links respectively, while the positive parameters *c*, $c^{+}$, and $c^{-}$ can be further used to modulate the average structural (*c*) or regulatory positive ($c^{+}$) and negative ($c^{-}$) degree of the structural links (number of regulator nodes).

The triadic interactions can activate or deactivate the structural links giving rise to triadic percolation. In triadic percolation, the activity of structural links that controls the network connectivity is determined by the triadic interactions that regulate them. Conversely, the activity of the nodes is dictated by the network connectivity via the percolation process.

Triadic percolation is defined as follows. At t=0, all structural links are active. For t≥1, the dynamics is given by a simple 2-step iterative algorithm:

- Step 1: Given the configuration of activity of the structural links at time t−1, nodes are considered active if they belong to the largest connected component of the structural network. Otherwise the nodes are considered inactive.
- Step 2: Given the set of all active nodes obtained in Step 1, all the links that are connected at least to one active negative regulator node and/or that are not connected to any active positive regulator node are deactivated. The remaining links remain intact only with probability *p*.

At each time step *t*, the state of the structural network is given by the binary vector s=s(t), of elements $s_{i}(t)$ indicating whether node *i* is active ($s_{i}(t)=1$) or inactive ($s_{i}(t)=0$) at time *t*. The size of the giant component *R* indicates the fraction of nodes in the giant component, which in triadic percolation is time dependent. Note that in triadic percolation the dynamics is deterministic for p=1, while for 0<p<1 the dynamics is stochastic, i.e., the triadic interactions do not fully determine the dynamical process. Thus, *p* acts as the control parameter of the dynamics.

It follows that spatial triadic percolation includes both quenched and annealed disorder. The quenched disorder is determined by the structure of the higher-order network with triadic interactions, while *p* drives the annealed disorder and dictates the randomness of deactivation events.

Triadic percolation in spatial networks generates topological patterns of the giant component with nontrivial dynamical evolution. These patterns and their dynamics depend significantly on the control parameter *p* that determines the highly nontrivial phase diagram of triadic percolation in spatial networks, as we discuss below.

## Emergence of triadic percolation patterns

Typically, in percolation theory, the percolation transition is described by monitoring the size *R* of the giant component as a function of the control parameter *p*. In spatial triadic percolation, we see that this measure fails to capture the complex topological patterns, called triadic percolation patterns, acquired by the giant component as long as the range of the structural and regulatory interactions are local. Hence, we need to develop and use additional quantitative methods to classify these patterns and encode their information content.

**Fig. 2.**
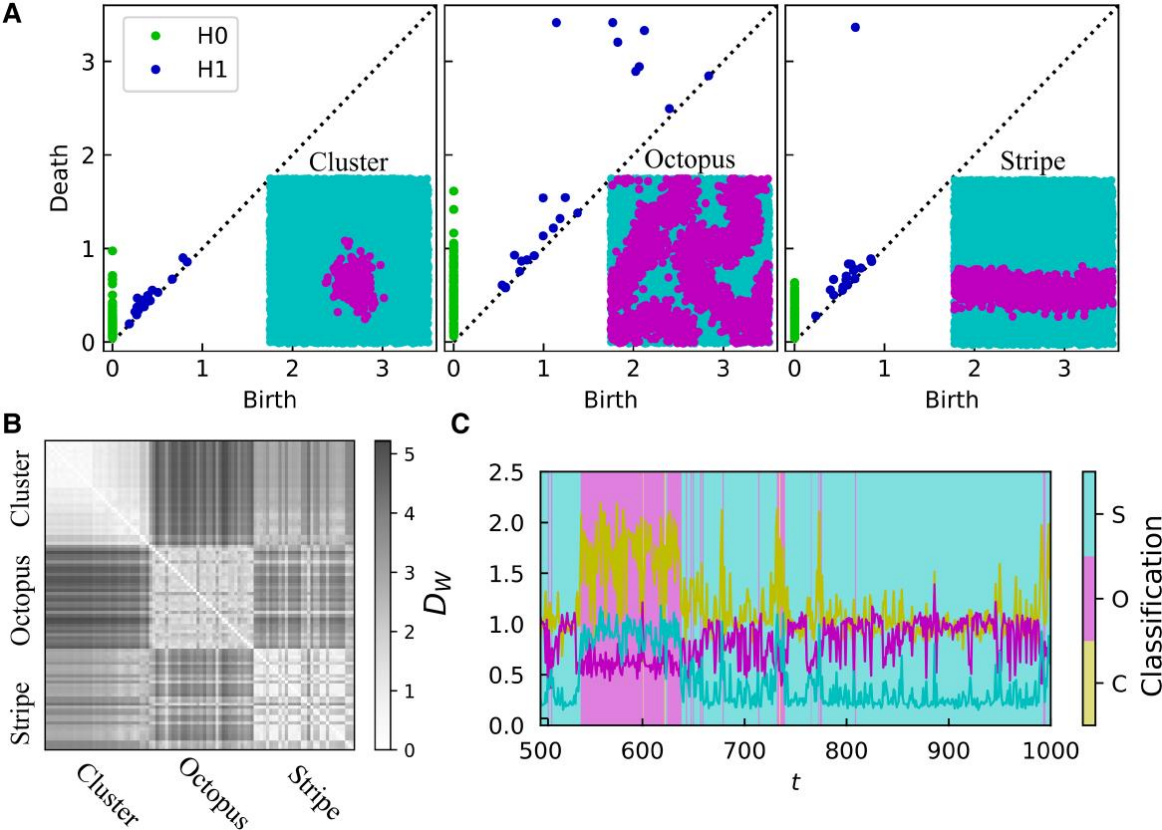
Topological classification of the triadic percolation patterns. A) The 0-homology ($H_{0}$) and 1-homology ($H_{1}$) persistence diagrams corresponding to an exemplary *Cluster* (C), *Octopus* (O), and *Stripe* (S) patterns, as shown by the insets, where turquoise (pink) dots stand for inactive (active) nodes. Major differences in the persistence diagrams of $H_{0}$ and $H_{1}$ can be observed. B) Pattern dis-similarity is measured with the Wasserstein distance $D_{W}$, here shown between template patterns corresponding to the Cluster, Octopus, and Stripe patterns as indicated by the labels. To perform the classification, 33 templates of each pattern class have been considered. Octopus patterns show larger within-class topological variability. C) Illustrative pattern classification for an exemplary time-series. First, the distance of each state s(t) to each pattern class *P*, P∈{C,O,S} is taken to be the minimum $D_{W}$ distance between s(t) and the template states of each pattern class (data lines). Then, the triadic percolation pattern at time step *t* is assigned the closest template pattern (as indicated by the shaded background areas). Here the triadic percolation results are obtained for p=0.8, $N=10^{4}$, $c^{+}=c^{-}=0.2$, $d_{0}=d_{r}=0.25$, c=0.4, ρ=100.

Visual inspection of the spatial distribution of the giant component induced by spatial triadic percolation when $c^{+}=c^{-}$ and $d_{0}=d_{r}$ define short range spatial interaction, suggests the emergence of three qualitatively different types of patterns as shown in Fig. 2:

1. Small clusters of active nodes, of relatively small size.
2. Octopus, tubular-like shapes predominantly formed by wide lanes that cross the borders (potentially more than once) and may also form inner loops.
3. Stripes, either horizontal or vertical, that reach the borders forming closed loops (due to the periodic boundary conditions) and are of similar width to the octopus patterns. Multiple stripes may also emerge.

The emergent patterns share a strong spatial structure, with separated active (high density of active nodes) and inactive (null density) areas, leading to a heterogeneous distribution of activity. For a discussion of the patterns observed for more general choices of the parameter values see Supplementary material.

### TDA classification of triadic percolation patterns

To classify the triadic percolation patterns, we have considered their distinct topology. Even if clusters and stripes can be of similar or comparable size, the critical difference between them is that stripes reach the borders of the torus and close a loop: whereas clusters have no holes, stripes have one (per stripe in the pattern). Conversely, octopus patterns may present more than one macroscopic component (typically of different sizes) and at least one hole.

TDA, and specifically persistent homology (82, 83), is the ideal method to detect these differences. Persistent homology encodes a point cloud (in this case the triadic percolation patterns) into a simplicial complex and then tracks the *k*-homological classes. The *k*-homological classes define the topology of the simplicial complex and are in one-to-one correspondence with the independent *k*-dimensional holes: in dimension 0, these are connected components; in dimension 1, cycles (or loops), i.e. 1D holes; in dimension 2, 2D holes (as in a triangulation of a sphere), and so on. Here, we consider Vietoris–Rips complexes, built from the point cloud by connecting all points at a spatial (Euclidean) distance smaller than a certain value (radius) and by filling all the cliques of the resulting network. By increasing the radius (i.e. the maximum distance between nodes to be considered connected), the persistent homology diagram indicates the emergence and death of each homological class of the data (see Fig. 2A). The (dis-)similarity between two patterns can then be measured by means of the Wasserstein distance $D_{W}$ between their respective persistence diagrams (see Materials and methods section for details).

The distance $D_{W}$ between same-class patterns is much smaller than the between-class distance, as observed in Fig. 2B for hand-selected *template* patterns (n=33 of each class). This result quantifies the finding that emergent pattern classes present distinct homology. Notably, the Wasserstein distance also captures the larger variability within the octopus class, with larger $D_{W}$ values. Using the set of template patterns, we classified each emergent pattern into one of the three nominal classes by identifying the closest (smallest $D_{W}$) class to the pattern, as illustrated in Fig. 2.

In this manner, TDA can provide a quantitative classification of the topological patterns acquired by the giant component in spatial triadic percolation. Hence, the spatio-temporal dynamics of the giant component can be considered as a time-series of distinct topological patterns. Note however that, as we will reveal in the following, triadic percolation gives rise to a dynamics of triadic percolation patterns that is also largely affected by their geometry (for instance the position of the barycenter of the patterns).

### Information theory of triadic percolation patterns

The emergent patterns can be further investigated with information theory tools. In particular, for each class of triadic percolation patterns we study the distribution of the size *R* of the giant component, together with the permutation entropy *H* and the complexity *C* of the patterns. While *R* is the usual order parameter for percolation, the permutation entropy *H* and the complexity *C* were formulated originally for quantifying local spatial patterns on 2D images (74) and patterns (75, 76).

Permutation entropy *H* quantifies the “randomness” of the local spatial patterns by calculating the entropy of pixel permutations. Higher permutation entropy values indicate greater irregularity in the data. Complexity *C* provides additional information about the degree of correlational structure by considering larger spatial patterns of motifs. A high permutation complexity value indicates the presence of diverse, less repetitive patterns of the data. Complexity will reach a minimum on either completely random patterns or regular patterns, and a maximum on patterns with “hidden” structure (84–86).

**Fig. 3.**
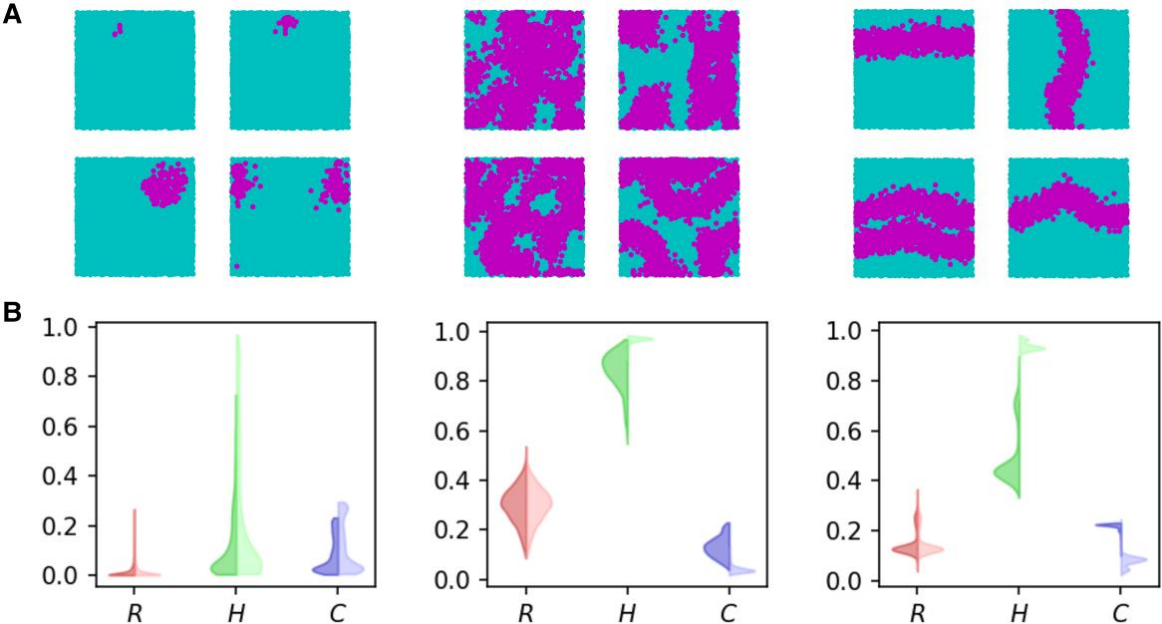
Examples of spatial triadic percolation patterns and their properties. A) Examples of the triadic percolation patterns: Small clusters (first column), Octopus (second column), and Stripes (third column). Active nodes are highlighted in pink and inactive nodes are in turquoise. B) Quantitative characterizations of the triadic percolation patterns. The size *R*, permutation entropy *H*, and complexity *C* of patterns are shown to reveal important quantitative differences between patterns. For each pattern, a surrogate random pattern was created with the same size *R* and randomly selected active nodes. The distributions of *R*, *H*, *C* for each pattern class are shown by two-sided violin plots, where the left-hand side corresponds to the spatial triadic percolation patterns, and the right-hand side to the surrogate random patterns. The values of the entropy *H* and the complexity *C* are obtained by embedding active nodes into a 30×30 grid with sliding partitions of size $d_{x}=2$ and $d_{y}=2$ (see details in Materials and methods section). The spatial network with triadic interactions is obtained by a realization of the model with parameter values $N=10^{4},c^{+}=c^{-}=0.2$, $d_{r}=d_{0}=0.25$, c=0.4. The triadic percolation patterns are derived (for classification details see Fig. 2) from simulations with p=0.1,0.2,…1.0, each including 500 time-steps (after a transient period of 500 steps).

In Fig. 3B, we report the distributions of *R*, *H*, and *C* for each pattern class. As already hinted by qualitative observations, the three triadic percolation pattern classes differ on the average size $\bar{R}$, with clusters being typically small, octopus spanning up to half of the nodes of the network, and stripes presenting intermediate values. However, *R* is not enough to distinguish individual patterns, as evidenced by the overlap in the distribution of *R* values between pattern classes. For instance, a stripe pattern may include more than one stripe, resulting in a stripe pattern with a relative large size, and similarly cluster patterns may be relatively large, i.e. of similar size to the typical stripes (for instance, see Fig. 3B third column).

Quantitatively, the topology or the complexity of the pattern can neither be distinguished by means of the size *R* alone. In particular, random patterns with no spatial structure can emerge with any given value of *R* (on nonspatial networks). On the contrary, the permutation entropy *H* and complexity *C* provide extra information on the rich spatial organization of the patterns, which is remarkably different from that of random patterns. To quantify this finding, in Fig. 3B, we compare the actual distribution of *H* and *C* (left-hand side of each violin plot, darker color) with the distribution obtained from surrogate random patterns with the same size as each of the spatial triadic percolation patterns (right-hand side of each violin plot, darker color). As it can be observed, octopus and stripes are vastly different from random patterns, with (a) consistently lower permutation entropy *H*, (b) higher complexity *C*, and (c) wider distributions of *H* and, in the case of octopus, also wider distribution of *C*. Cluster patterns on the contrary are less different from surrogate random patterns, suggesting that the observed values of *H* and *C* are mostly driven by the pattern size *R* in the case of uniform clusters.

The three classes of triadic percolation patterns differ in terms of absolute values of entropy and complexity. Octopus patterns show on average the largest permutation entropy, followed by stripes, whereas clusters in general present low values of *H*. Regarding complexity, this is highest for stripes, followed by octopus, whereas clusters present typically small values but with large deviations. Thus, on this meso-scale description, stripes are the least random pattern.

Finally, we notice that not only the average value of *H* and *C* differs between pattern classes, but their within-class distributions also show specific properties. Octopus show the largest variation of *H* and *C*, evidencing large within-class variability. For stripes, two well-defined maxima of *H* are observed, corresponding respectively to single and double stripes. Remarkably, these barely differ in terms of complexity, which is in strong contrast with surrogate random patterns of the same size, for which the two maxima are observed again. Thus, all stripes are similar in terms of complexity, whereas the variability in octopus does lead to different complexity.

## Dynamics of triadic percolation patterns

**Fig. 4.**
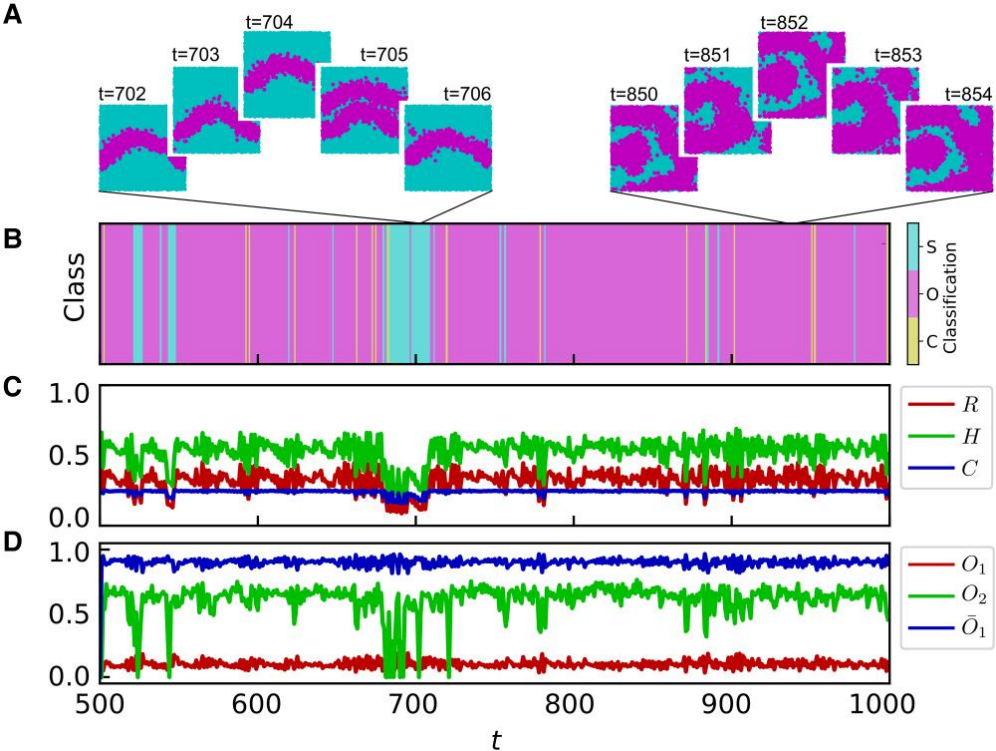
Exemplary intermittent time-series of spatial triadic percolation patterns. A–D) Time-series of triadic percolation patterns observed for p=0.7 on the same network realization as Fig. 2. A) shows the spatial triadic percolation patterns at exemplary time steps. B and C) show respectively the pattern classification (cyan for stripes S, pink for octopus O, and yellow for clusters C), the size of the giant component *R*, the entropy *H* and the complexity *C* of the triadic percolation patterns a function of time. To illustrate the temporal dynamics of the triadic percolation patterns (D), we monitor the overlap $O_{\tau }$ between active patterns at lag τ=1,2 (leading to $O_{1}$ and $O_{2}$), and the overlap ${\bar{O}}_{1}$ between the active and inactive patterns at lag τ=1.

Triadic percolation displays a nontrivial temporal organization of the dynamics of its emergent topological patterns. The topology of the triadic percolation patterns displays metastability, with patterns belonging to the same topological class persisting for varying lifetimes depending on both the quenched and the annealed disorder of the triadic percolation model. In particular, intermittent time-series of the triadic percolation patterns occur for a wide range of parameters values. For instance, in Fig. 4, we show a time-series in which both octopus and stripes occur in an intermittent and dynamic fashion. Videos of exemplary time-series of triadic percolation patterns are included in the Supplementary Videos.

The temporal behavior of the fraction of nodes in the giant component *R*, and of the information theory measures (entropy *H* and complexity *C*) capture important aspects of the dynamics of triadic percolation (see Fig. 4C). However, there are several nontrivial spatio-temporal effects which go beyond the information that can be extracted from *R*, *H*, and *C* alone.

The emergence of time-varying triadic percolation patterns is a direct consequence of local connectivity and also local positive and negative regulation. Local negative regulation leads to emergent self-inhibition in the macroscopic scale, whereas local positive regulation implies that activity can only emerge on the neighborhood of previously active regions. Together, these mechanisms create and effect surface tension, and give rise to the emergent macroscopic patterns of different shapes, see Supplementary material for further details. As a consequence, we observe that in the deterministic case stripes always emerge through surface minimization. Thus on a torus constructed from a rectangular map (rectangular torus), stripe patterns only occur along the shorter dimension of the rectangular torus (see Supplementary material).

Crucially, this mechanism induces an intrinsically dynamic nature of the triadic percolation patterns s(t). Moreover, given that positive regulation occurs on a local scale, the active pattern at time t+1, s(t+1), can only appear on the neighborhood of the active pattern at time *t*, s(t). In combination with effective self-inhibition, this causes the tubular geometry of the stripe and octopus patterns.

The combination of self-inhibition and border-excitation also induces the temporal dynamics of the patterns, characterized by two dominant mechanisms at short-time scales: short-time-blinking (ST-blinking) and diffusion. ST-blinking consists of the alternation of complementary patterns such that s(t+1)≈1−s(t). ST-blinking occurs for large patterns (typically octopus) such that the pattern and its neighborhood encompass the whole structural network. Diffusion, on the contrary, occurs for smaller patterns (small clusters) whose neighborhood does not cover all the inactive region. In this case, the active pattern may not return after two steps, as more options are available, the probability of each one depending on the specific local fluctuations of connectivity.

ST-blinking and diffusion can be quantified by the overlap between patterns at different time-steps, defined as $O_{\tau }=\sum_{i}s_{i}(t)s_{i}(t+\tau )/\sum_{i}s_{i}(t)$, where $O_{\tau }(t)≃1$ indicates that patterns s(t) and s(t+τ) are similar, whereas values close to 0 indicate that they are significantly different. In particular, in Fig. 4D, we show the overlap between consecutive active patterns, $O_{1}=O_{1}(t)$, and between patterns separated by two steps, $O_{2}=O_{2}(t)$, and the overlap between the active pattern at time *t* and the inactive pattern at time t+1, ${\bar{O}}_{1}=\sum_{i}s_{i}(t)(1-s_{i}(t+1))/\sum_{i}s_{i}(t)$. Due to emergent self-inhibition, $O_{1}$ is always low and ${\bar{O}}_{1}$ high, whereas $O_{2}$ distinguishes between ST-blinking and diffusion: it is large for ST-blinking, and small for diffusion. In the exemplary case of Fig. 4, we observe in general high $O_{2}$, indicating a domination of ST-blinking, although it intertwines with diffusion when stripes emerge (see Fig. 4A and D around t=700). In general, we have found that octopus patterns blink on short-time scales, and clusters diffuse, whereas stripes show a mixed behavior, as shown in Supplementary material.

The microscopic organization of the structural and regulatory networks shapes the dynamics of the patterns, as easily observed for stripes. Assuming fully homogeneous local connectivity, 1D diffusion (akin to a random walker on a 1D lattice) would be the expected behavior for stripes: a horizontal stripe may move up or down, with equal probability. However, we observe a location-dependent asymmetry in the movement, that effectively traps the stripes into preferred locations. This asymmetry is induced by microscopic inhomogeneities or quenched disorder. The effect of quenched disorder is less evident for increased random damage, which adds annealed noise into the dynamics (see Supplementary material).

**Fig. 5.**
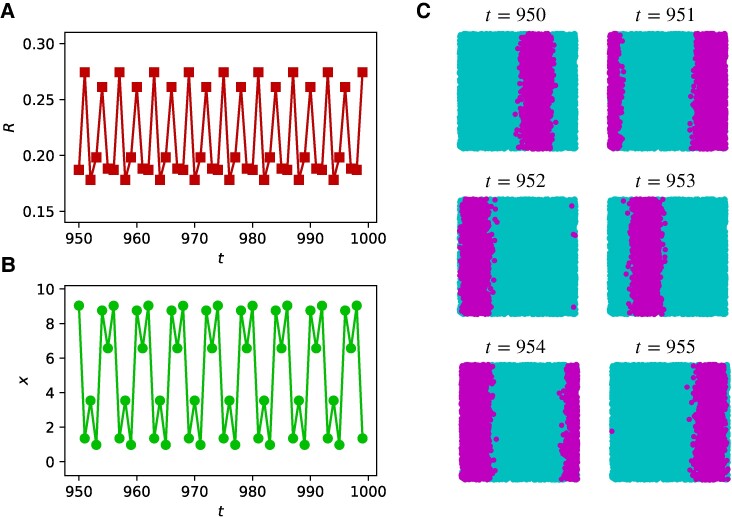
Blinking of stripe patterns. A) Relative size of the giant component *R* is shown a function of time *t*. B) The *x*-coordinate of the barycenter of the patterns is shown as a function of time *t*. C) Examples of stripe patterns. Active nodes are highlighted in pink and inactive nodes are in turquoise. All these measures clearly indicate the sustained blinking of the triadic percolation patterns, which in this case is a periodic dynamics with period 6. The spatial network is formed by $N=10^{4}$ nodes. The parameters for generating the spatial network with triadic interactions are $c^{+}=0.4$, $c^{-}=0.2$, $d_{r}=d_{0}=0.25$, c=0.4, p=1.

Finally, we note that triadic percolation patterns can blink, i.e. display sustained periodic dynamics, depending on the properties of the structural and regulatory networks (see Fig. 5). Our previous discussion accounted for the case of $c^{+}=c^{-}$. For illustration purposes, we now consider the case of strong positive regulation ($c^{+}=2c^{-}$) and the deterministic scenario (no random damage, p=1). More general cases where $c^{+}\neq c^{-}$ are discussed in the Supplementary material. In this case, stripes are the only observed pattern (as for $c^{+}=c^{-}$, p=1). For instance, in Fig. 5A, B, we show evidence of blinking with a period-6 dynamics for both *R* and the barycenter location *x*. This blinking behavior consisting of sustained periodic dynamics of the triadic percolation patterns is also validated by the inspection of the actual triadic percolation patterns shown in Fig. 5C.

These results allow us to draw a general interpretation of the observed spatio-temporal phenomenology. Triadic percolation on spatial networks leads to complex spatio-temporal dynamics. Two spatio-temporal scales emerge. Spatially, the patterns are heterogeneous, with the emergence of well-defined active and inactive regions. At short-time scales, we observe a dynamics dominated by ST-blinking, for intermediate random damage and large patterns, and by diffusion, for large random damage and small (clusters) patterns. In the case of stripes, quenched disorder caused by random local connectivity breaks translational symmetry, and preferred locations for stripes emerge at the mesoscopic scale, leading to ST-blinking and in some cases to sustained periodic blinking of triadic percolation patterns, particularly for the deterministic (no random damage) case. The specific nodes becoming active on each appearance of the macroscopic stripe may vary and the barycenter shows small microscopic variations, but it remains contained within the width of a macroscopic stripe. In absence of sustained periodic blinking, a combination of random (annealed) damage and quenched disorder, caused by random local connectivity, deforms the emergent patterns on large temporal scales.

## Phase diagram of spatial triadic percolation

**Fig. 6.**
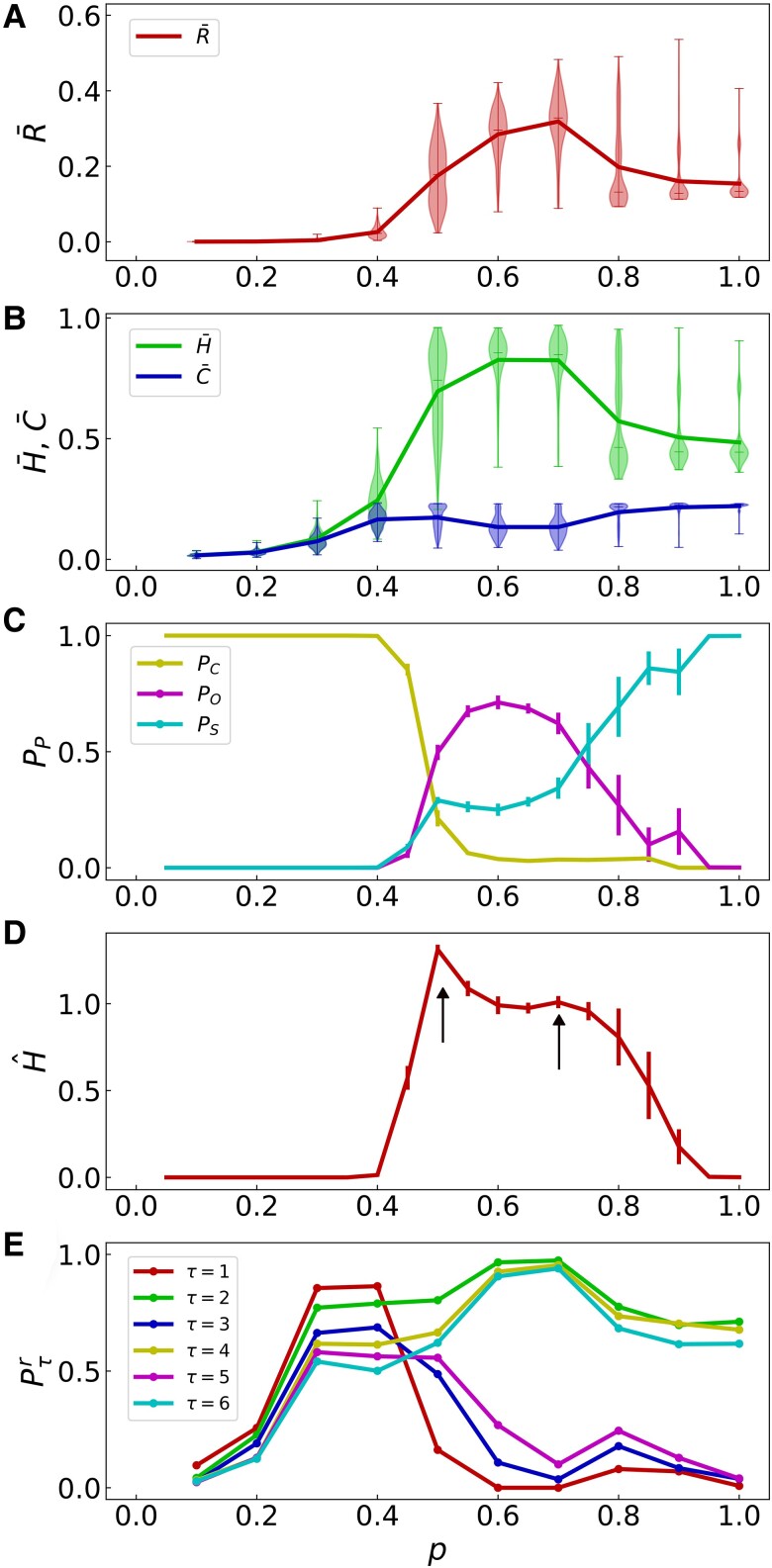
Phase diagram of spatial triadic percolation. A, B) The average size $\bar{R}$ of the giant component (A), the average values of the permutation entropy $\bar{H}$, and the complexity $\bar{C}$ of the triadic percolation patterns (B) are plotted as a function of *p*. C, D) The TDA classification is adopted to enrich the phase diagram with static and dynamical information about the topology of the patterns. The fraction of triadic percolation patterns classified as Clusters ($P_{C}$), Octopus ($P_{O}$), and Stripes ($P_{S}$) (C) and the entropy rate $\hat{H}$ of the pattern time-series (D) are plotted as a function of *p*. The black arrows indicate two local minima. E) Displays the probability $P_{\tau }^{r}$ of return after *τ* steps as a function of *p*, averaged over the time-series, revealing the nature of the short-time dynamics of the triadic percolation patterns. The spatial network with triadic interaction is formed by $N=10^{4}$ nodes and generated with parameter values $c^{+}=c^{-}=0.2$, $d_{r}=d_{0}=0.25$, c=0.4 in all panels. A, B, E) are obtained from the same data corresponding to 500 steps of the triadic percolation dynamics. The entropy $\bar{H}$ and the complexity $\bar{C}$ are obtained by embedding active nodes into a 30×30 grid with sliding partitions of size $d_{x}=2$ and $d_{y}=2$. The accompanying violin plots show the distribution of values of each variable. C, D) are obtained from the average of 10 different iterations of the triadic percolation dynamics, each including 1500 steps of the dynamics. The curves show the average values, and the errorbars the standard deviation among network realizations. Data were considered after a transient of 500 steps in all cases.

The phase diagram of triadic percolation, studied as a function of the control parameter *p* (see Fig. 6) reflects its complex spatio-temporal dynamics. In order to investigate this phase diagram, we first monitor the relative size of the giant component *R* as a function of *p* (see Fig. 6A). The first important observation that we make is that on average, the relative size of the giant component $\bar{R}$ displays a maximum and is hence not monotonically increasing with *p* as in standard percolation. Moreover, the relative size of the giant component is roughly bounded by half of the nodes of the network. This is a consequence of the competition between local positive and negative regulation, given that active nodes at time t+1 are limited to the area nearby (but, crucially, excluding) the active pattern at time *t*. As we have discussed extensively in the previous sections, however, the value of *R* does not provide detailed information on the triadic percolation patterns. To investigate the nature of the triadic percolation patterns, we monitor the average of the information theory measures *H* and *C* (given by $\bar{H}$ and $\bar{C}$) as a function of *p* (see Fig. 6B). Most relevantly we find that also the average permutation entropy $\bar{H}$, has a maximum for intermediate values of *p* indicating more random patterns. The quantities R,H and *C* have the advantage of being unsupervised quantities, we note however that, due to their large within-class variability (as reported in Fig. 3), R,H,C have limited ability to detect the regions of the phase diagram with distinct topological organization (see Fig. 6A, B). The phase diagram of triadic percolation is therefore enriched by considering the results of TDA analysis (see Fig. 6C). The TDA analysis reveals three regions of the phase diagram, each dominated by a single type of pattern, although values of *P* with significant coexistence of patterns are observed. For small values of *P* (large probability of random edge damage), low-activity patterns, i.e. clusters, are most likely. On the other hand, for large values of p≃1 (strongly suppressed random edge failure), most regulation links are active, and the combination of local positive and negative regulation results in stripe patterns due to the border-minimization effect (see Supplementary material). For intermediate values of *P*, the octopus topology emerges as the most likely. The three TDA-detected regions are observed for ranges of *P* values that depend on the other parameters of the model, although octopus and stripe patterns require that the active cluster is percolating, ensuring the connectivity of the giant component. Moreover, stripes always emerge for P=1 (no random damage).

As triadic percolation is a dynamical process, the phase diagram is not complete if we do not provide information about the dynamical nature of the time-series of patterns as a function of the control parameter *p*. To this end, we have considered two quantities that capture the temporal dynamics of the macroscopic patterns: the entropy rate $\hat{H}$ of the pattern time-series, and the return probability $P_{\tau }^{r}$ of the overlap $O_{\tau }$ time-series defined in a previous section. These quantities probe the temporal dynamics of the system at different scales: the entropy rate $\hat{H}$ captures the long-term behavior (as revealed by Fig. 5) and $P_{\tau }^{r}$ quantifies the short-time dynamics typically observed, and can be used to classify diffusion and ST-blinking of patterns in time.

The entropy rate $\hat{H}$ (see Materials and methods for details) quantifies the information content of the time-series of patterns as derived from the TDA classification analysis (Fig. 6C). Therefore the complex triadic percolation dynamics is codified by the single time-series of the pattern classification (a series of letters indicating the three distinct type of topological patterns –clusters, octopus and stripes). Very predictable time-series have low entropy rate, highly unpredictable ones have high entropy rate. Hence the entropy rate $\hat{H}$ can be used to probe intermittent time-series of patterns and can be related to the complexity of the process (87) (see Supplementary material for details). Interestingly the entropy rate $\hat{H}$ presents evidence for a dynamical phase transition at $p=p_{c}≃0.4$ (see Fig. 6D). For $p<p_{c}$ only one type of pattern is observed (clusters) and the entropy rate is zero, i.e. $\hat{H}=0$; beyond this phase transition different types of patterns coexist and thus we have $\hat{H}>0$, while for p≃1 the entropy rate is again zero, i.e. $\hat{H}=0$ and only stripe patterns are observed. Moreover the entropy rate $\hat{H}$ displays two local maxima corresponding to values of *p* with more diverse compositions of pattern types. The first local maximum (smaller *p*) is higher due to the coexistence of the three pattern classes (clusters, octopus, and stripes) while in correspondence of the second local maximum (larger values of *p*) we observe coexistence only of two pattern classes (octopus and stripes).

The second measure we introduce is the return probability $P_{\tau }^{r}$ (see Materials and methods section for details) that measures the probability that a pattern re-occurs (or returns) after *τ* steps, and is analogous to the return probability on a diffusion problem (88). Hence this measure differs from the first not only because it probes the dynamics at smaller time-scales but also because it is very sensible to the geometrical details of the patterns (typically having the same topological classification). As observed in Fig. 6E, $P_{\tau }^{r}$ follows a nontrivial trend in the three regions of the phase diagram dominated by the three different topologies (Fig. 6A). For even *τ* values, there is a maximum in the octopus-dominated region (intermediate *p* values), with sustained high values in the stripes region (large *p* values) and minimum values for the cluster-dominated (small *p* values) region. For odd *τ* values we find low values on the octopus and stripes region due to the strong ST-blinking effect. Thus, octopus patterns and stripes have a larger probability of re-occurrence at short-time scales (associated with a ST-blinking dynamics), than clusters.

The phase diagrams derived from the temporal dynamics ($\hat{H}$ and $P_{\tau }^{r}$) follow closely those derived from the spatial organization (pattern occurrence, $\bar{R}$, $\bar{H}$, and $\bar{C}$), indicating that each pattern class not only presents distinct geometry and homology, but also a characteristic macroscopic evolution of the patterns.

As we have seen, the phase diagram of triadic percolation provides a comprehensive understanding of triadic percolation. In particular, this phase diagram covers the topology, the information content and the dynamics of this very rich dynamical process going beyond a description of triadic percolation based exclusively on the size of the giant component. For a discussion of the phase diagram depending on the other parameters of the model see the Supplementary material.

## Conclusions

In summary, triadic interactions are fundamental higher-order interactions present in a variety of complex systems, ranging from brain networks to ecosystems, that can dramatically change the properties of percolation, as captured by triadic percolation. Triadic percolation on random graphs (12) has been previously shown to lead to a nontrivial dynamics of the standard order parameter, given by the fraction of nodes in the giant component. Here, we show that, on spatial networks, triadic percolation not only leads to a time-varying fraction of nodes in the giant component, but displays remarkably different spatio-temporal patterns of the giant component. Specifically, in spatial triadic percolation the topology itself of the giant component changes in time. In order to investigate the spatio-temporal properties of triadic percolation we combine network science, TDA, information theory, and the theory of nonlinear dynamical systems to describe this remarkable critical phenomenon. The giant component displays patterns that are classified through persistent homology into three different classes: small clusters (formed by a localized scattered set of points), octopus patterns (patterns with nontrivial persistent diagram), and stripes (patterns of points going around the torus). These emergent spatial triadic percolation patterns are remarkably different from random patterns, as quantified by our information theory analysis, which reveals the lower entropy and larger complexity of these patterns with respect to random unstructured patterns. These patterns have a very nontrivial dynamics revealing that the giant component of triadic percolation has a topology that can change significantly over time. We show that for some parameter values the time-series of patterns displays intermittency between different topological classes. Moreover, in the case of stripes, we provide evidence of blinking behavior with the barycenter of the giant component oscillating periodically. These findings are summarized using a phase diagram of triadic percolation, indicating the regions where patterns of a given type are more likely to occur. The phase diagram also shows how statistical, information theory, and temporal observables of the complex spatio-temporal dynamics of the giant component change as a function of the control parameter leading to a comprehensive understanding of these complex dynamics.

The observed spatio-temporal modulation of the topology and geometry of the giant component opens new perspectives in percolation theory and its applications. As giant components changing dynamically in time are observed in a large variety of real systems, these findings have the potential to transform our theoretical understanding of these systems. Our hope is that this theoretical framework will be relevant for developing specific models in neuroscience and climate and for the formulation of inverse algorithms to analyze higher-order network data.

## Materials and methods

### Spatial higher-order networks with triadic interactions

We consider spatial higher-order networks with triadic interactions embedded in a 2D square of size *L* with periodic boundary conditions (a torus). The density of nodes is indicated by *ρ*, with *ρ* fixed as 100 nodes per unit square in all simulations of this article. The structural networks $G_{s}=(V,E)$ contain edges drawn randomly between each pair of nodes with probabilities decaying exponentially with their Euclidean distances. Specifically, a pair of nodes *i* and *j* are connected with probability


(1) $$P_{ij}=ce^{-d_{ij}/d_{0}},$$

where $d_{ij}$ denotes the Euclidean distance between nodes *i* and *j*, and $d_{0}$ denotes a typical length of structural links. The average structural degree ⟨k⟩ is controlled by 0<c≤1. The spatial regulatory network is generated as follows. First, we define the coordinate of a structural link ℓ as the midpoint of its two end nodes. Then, a positive or negative regulatory interaction between a node *i* and a structural links ℓ is drawn with probability ${\hat{P}}_{i\ell }^{+}$ and ${\hat{P}}_{i\ell }^{-}$, respectively. The probabilities ${\hat{P}}_{i\ell }^{+}$ and ${\hat{P}}_{i\ell }^{-}$ are given by


(2) $$\begin{array}{rl} {\hat{P}}_{i\ell }^{+} & =c^{+}e^{-d_{i\ell }/d_{r}^{+}},\\ {\hat{P}}_{i\ell }^{-} & =c^{-}e^{-d_{i\ell }/d_{r}^{-}}, \end{array}$$

where we exclude the possibility of conflicting regulatory interactions. In otherwords, we impose that if a node is a positive regulator of a link it cannot be simultaneously a negative regulator of the link. In the main, we focus on the scenario where positive and negative regulations have the same length scale, i.e., $d_{r}^{+}=d_{r}^{-}=d_{r}$. In the Supplementary material, we discuss the general scenario of $d_{r}^{+}\neq d_{r}^{-}$. In Eq. 2, $d_{r}$ defines the typical length of regulatory interactions, and $c^{+}$ and $c^{-}$ (with $c^{+}>0,c^{-}>0$ and $c^{+}+c^{-}\leq 1$) control the average number of nodes that regulate a link positively or negatively. For $d_{r}\to \infty$ and $d_{0}\to \infty$, the spatial network with triadic interactions reduces to the Erdös–Renyi network with random triadic interactions that is defined and studied in Ref. (12).

### TDA-based classification

The spatial triadic percolation patterns are here classified by means of topological data analysis (TDA). TDA allows us to identify a pattern’s shape and its invariant topological properties, with moderate noise tolerance (82, 89–91). The TDA of a set of points proceeds by first generating a simplicial complex that represents the data at different values of a *filtration* or threshold parameter $f_{s}$, and then evaluating the homology classes of the simplicial complex as function of $f_{s}$. A simplicial complex is a finite collection of simplices *K* such that (i) every face of a simplex in *K* also belongs to *K* and (ii) for any two simplices $\sigma_{1}$ and $\sigma_{2}\in K$, if $\sigma_{1}\cap \sigma_{2}\neq \emptyset$, then $\sigma_{1}\cap \sigma_{2}$ is a common face of both $\sigma_{1}$ and $\sigma_{2}$. A *d*-simplex is the convex hull of d+1 points: a 0-simplex is a point, a 1-simplex is an edge, a 2-simplex is a triangle and so on. Here, we consider the Vietoris–Rips filtration method to build simplicial complexes that represent the data. Given a pattern of active nodes at time *t*, s(t), the corresponding Vietoris–Rips complex is the filtered complex $VR_{s}(s(t))$ that includes all *δ*-simplices, δ≤d, such that all pairwise distances between the nodes in the simplex are equal or less than $f_{s}$, that is $VR_{s}(s)=\{[v_{0},\ldots ,v_{n}]\forall \;i,j\;d(v_{i},v_{j})\leq f_{s}\}$. The *k*-homological classes (82, 83) of a simplicial complex are in one-to-one correspondence with its independent *k*-dimensional holes: in dimension 0, these are connected components, in dimension 1, cycles (also called loops), in dimension 2, 2D holes (like in a triangulated sphere), and so on. Persistent homology tracks the homology classes as the filtration parameter $f_{s}$ increases and detects which topological features persist across different scales (92). The filtration values at which each homological class emerges (birth) and disappears (death) can be recorded on a persistence diagram PD (showing the death value as a function of the birth value) and characterize a given shape or pattern. Points further from the diagonal mark features that survive for long filtration intervals. PDs of different patterns can be compared by measuring the distance between them. Here, we considered the Wasserstein distance, which matches pairs of points between the two diagrams and measures the $L^{p}$ distance between them. Points that cannot be matched to a point in the other diagram are matched to the diagonal.

The patterns were classified into Clusters (C), Octopus (O), and Stripes (S) using persistence homology. First, a set of representative template patterns (33 for each pattern class) were manually identified (an example for each class is given in the insets of Fig. 2A), and PDs were obtained for each one (Fig. 2A). Secondly, persistence homology was applied to each pattern s(t) of a given simulation of the system. The Wasserstein distance between the PD of s(t) and all template PDs was measured, and state s(t) was assigned the pattern class of the closest template (Fig. 2D). For the VR filtration, we consider simplices up to d=2, and the persistence diagrams included the 0-($H_{0}$) and 1-($H_{1}$) dimensional holes. The total Wasserstein distance was defined as the sum of the $H_{0}$- and $H_{1}$-associated distances. All TDA analyses were performed using the giotto-tda python library.

### Entropy and complexity

The structural and information theory properties of the spatial triadic percolation patterns include their permutation entropy and statistical complexity. The measure of permutation entropy was first proposed by Bandt and Pompe (75) to measure the complexity of 1D time-series. The approach was generalized to higher-dimensional data and an additional measure of complexity called López-Ruiz–Mancini–Calbet (LMC) complexity was proposed. The LMC complexity provides structural information that is not included in the entropy measure (76, 93). The permutation entropy is calculated based on the permutation of local partitions. Consider a 2D pattern that is represented by a matrix. We consider all local partitions of size $\left(d_{x},d_{y}\right)$, i.e., submatrices of size $d_{x}$ by $d_{y}$, where $d_{x}$ and $d_{y}$ are called embedding dimensions (74). Let us consider a simple case where $d_{x}=d_{y}=2$. Thus, each submatrix can be written as


(3) $$A=\left[\begin{array}{cc} a_{0} & a_{1}\\ a_{2} & a_{3} \end{array}\right].$$

Reshaping the submatrices to 1D vectors, we can categorize them into different ordinal patterns. For instance, pattern $\pi_{1}=(0,1,2,3)$ denotes all submatrices in which $a_{0}<a_{1}<a_{2}<a_{3}$ and pattern $\pi_{2}=(1,0,2,3)$ denotes all submatrices in which $a_{1}<a_{0}<a_{2}<a_{3}$. There are in total $(d_{x}d_{y})!=24$ ordinal patterns in this example. Thus, we can define the distribution of ordinal patterns with given embedding dimensions $d_{x}$ and $d_{y}$. The probability P(π) of having an ordinal pattern *π* is calculated by


(4) $$P(\pi )=\frac{\text{Number of submatrices that have pattern}\;\pi }{\text{Total number of submatrices of size}\;d_{x}\times d_{y}}$$

and the permutation entropy *S* is defined as


(5) $$S[P]=-\sum_{\pi }p(\pi )\ln\;p(\pi ).$$

The measure *H* used above is the normalized permutation entropy defined as


(6) $$H[P]=\frac{S[P]}{S_{\max}}=\frac{1}{(d_{x}d_{y})!}S[P].$$

The permutation entropy quantifies the amount of “information” in the patterns. The statistical complexity *C* composites the measure of information *H* and the measure of “disequilibrium” *Q* (93):


(7) $$C[P]=Q[P,P_{e}]H[P].$$

The disequilibrium $Q[P,P_{e}]$ is defined as the extensive Jensen–Shannon divergence that quantifies the distance between the pattern distribution P(π) and the uniform distribution $P_{e}$ (84);


(8) $$Q[P,P_{e}]=\frac{S[(P+P_{e})/2]-S[P]/2-S[P_{e}]/2}{Q_{0}},$$

where $Q_{0}$ is a normalization constant. Thus, the complexity *C* will reach zero at the extremes of the ordered pattern (H=0) and completely random pattern (Q=0). Note that *C* is not a trivial function and *H*. For a given *H*, there exists a corresponding range of *C* that provides additional structural information (74, 85). To calculate the entropy and complexity of patterns formed by active nodes, we transformed the activity pattern with a 2D density distribution over an M×M grid, over which we measure the permutation entropy *H* and complexity *C* of this matrix (94).

### Temporal analyses

The temporal dynamics of the emergent dynamics of triadic percolation is quantified by means of the entropy rate $\hat{H}$ of the pattern time-series x(t) derived from the TDA classification. That is, x(t) is the categorical time series indicating the macroscopic state of the system as Cluster (C), Octopus (O), or Stripe (S). To measure the entropy rate of a time series, we consider motifs or words of increasing length $\hat{L}$, and measure the relative count ${\tilde{\;p}}_{i}$ of each word *i* of such length. The entropy rate of $\hat{L}$-words is defined as (87):


(9) $$\hat{H}(\hat{L})=-\frac{1}{\hat{L}}\sum_{i}{\tilde{\;p}}_{i}\log_{2}\;{\tilde{\;p}}_{i}.$$

The entropy rate $\hat{H}$, is obtained in the limit of infinite word length, i.e.


(10) $$\hat{H}=\lim_{\hat{L}\to \infty }\hat{H}(\hat{L}).$$

Here in order to estimate this limit, we take the usual approach of extrapolating the linear trend of $\hat{H}(\hat{L})$ vs. $1/\hat{L}$ for $\hat{L}=1,\;2,\;4$, as detailed in the Supplementary material (see Supplementary material). Results shown in the main text are averaged over 10 time-series encoding the topology of 10 realizations of the triadic percolation dynamics.

At short-time scales, the dynamics is investigated by measuring the overlap between patterns at different time-steps, defined as


(11) $$O_{\tau }(t)=\frac{\sum_{i}s_{i}(t)s_{i}(t+\tau )}{\sum_{i}s_{i}(t)}.$$

$O_{\tau }(t)≃1$ indicates that the states s(t) and s(t+τ) are similar, whereas values close to 0 indicate significantly different states. To discriminate whether two states are *macroscopically* equivalent, we have set an adaptive threshold $\alpha^{\prime }$ on $O_{\tau }$, such that states *t* and t+τ are macroscopically equivalent if $O_{\tau }(t)>\alpha^{\prime }$ with


(12) $$\alpha^{\prime }=\frac{\alpha }{T}\sum_{t^{\prime }}O_{\tau }(t^{\prime }),$$

where α=0.8 is the baseline threshold. Here, $\alpha^{\prime }$ is given by the baseline threshold *α* rescaled by the average overlap $O_{\tau }$ of the time-series to account for the large fluctuations in the fraction of active nodes in the triadic percolation patterns. We thereby described each time-series by the binary variable ${\tilde{O}}_{\tau }(t)$ indicating whether the patterns at time *t* and time t+τ are macroscopically equivalent. Note that this analysis disregards the shape of the patterns. The variation of the overlap ${\tilde{O}}_{\tau }(t)$ in time is measured by the return probability $P_{\tau }^{r}$ (88, 95), that indicates the probability that the system returns at time t+τ to the same state it was at time *t*, namely $P_{\tau }^{r}=\sum_{t}{\tilde{O}}_{\tau }(t)/T$.

## Supplementary Material

pgae270_Supplementary_Data

## Data Availability

The codes used in this work are available at the GitHub repository Spatial-triadic-percolation.

## Preprints

This manuscript was posted on a preprint: arXiv:2311.14877.
