# Supplementary material for: Triadic percolation induces dynamical topological patterns in higher-order networks
Source: PNAS Nexus. 2024 Jul 9;3(7):pgae270. doi: 10.1093/pnasnexus/pgae270 (PMC11259606; doi:10.1093/pnasnexus/pgae270)
Supplement: pgae270_Supplementary_Data [file pgae270_supplementary_data.zip › PNASNEXUS-PNASNEXUS-2024-00029R-s05.pdf]

Supplemental Material on "Triadic percolation induces dynamical topological patterns in higher-order networks"

Ana P. Millán,[1] Hanlin Sun,[2] Joaquín J. Torres,[1] and Ginestra Bianconi[3,4]

[1] *Institute "Carlos I" for Theoretical and Computational Physics, and Electromagnetism and Matter Physics Department, University of Granada, E-18071 Granada, Spain*

[2] *Nordita, KTH Royal Institute of Technology and Stockholm University, Hannes Alfvns vg 12, SE-106 91 Stockholm, Sweden*

[3] *School of Mathematical Sciences, Queen Mary University of London, London, E1 4NS, United Kingdom*

[4] *The Alan Turing Institute, The British Library, London*

In this Supplementary Information, we provide additional material demonstrating the mechanisms for the emergence of short-time-blinking (ST-blinking) and diffusion of the triadic percolation patterns. We also provide background information on the Supplementary Movies. This discussion is relative to the extensive region of the parameter space in which sustained periodic blinking is not observed, such as for $c^+ = c^-$. Furthermore, we provide a discussion of the patterns observed for a wider range of parameter values exploring the dependence of the pattern as a function of $d_r = d_r^+ = d_r^-$ and as a function of d_r^+ and d_r^- chosen independently. Finally, we discuss the dependence on the parameters c^+ and c^-.

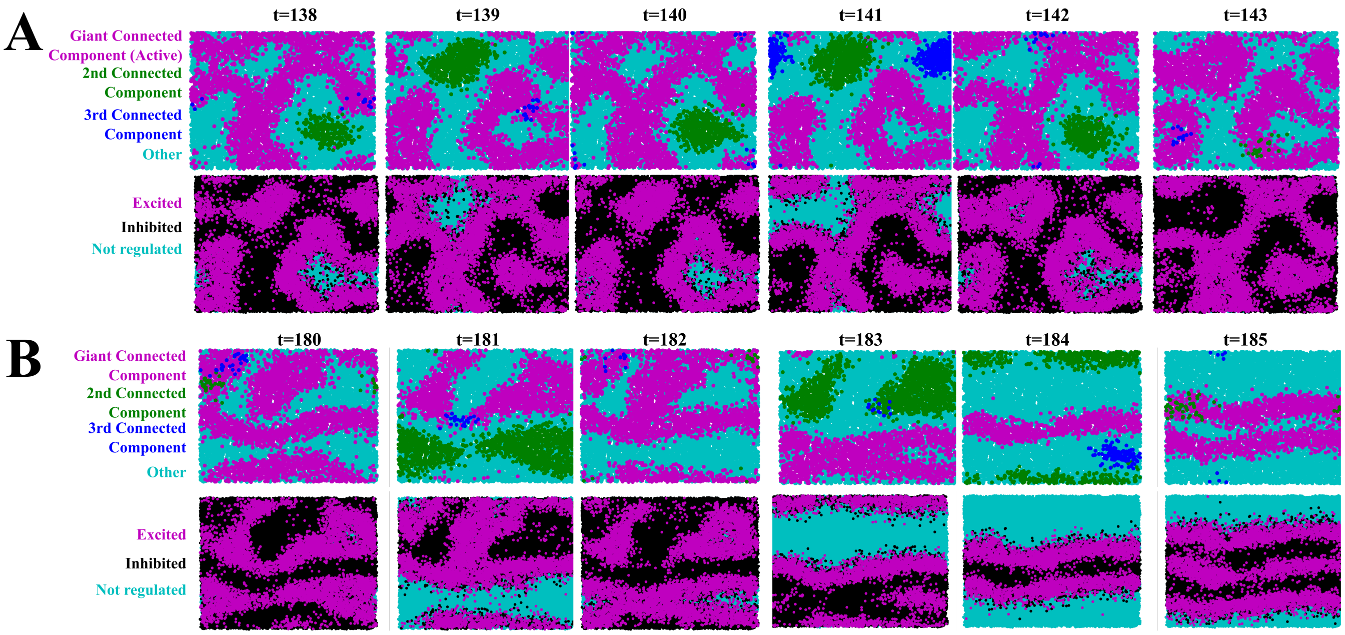

FIG. S-1. Mechanism underlying the emergence of spatial triadic percolation patterns. Two exemplary snapshots into the evolution of the triadic percolation patterns (**A, B**) are shown. The top panels (first and third row) show the states of the nodes, where active nodes (in the largest or giant connected component) are shown in pink as before, and we also show in green and blue the (inactive) nodes in the second and third largest connected components, for illustrative purposes. The remaining inactive nodes are shown in light blue as before. The bottom panels (second and fourth row) show the subsequent states of the structural links: up-regulated or excited (pink), down-regulated or inhibited (black), and not regulated (black). We do not show here random damage. Excited links are defined as those that are exclusively up-regulated, whereas inhibited links comprise all down-regulated links, regardless of whether they are up-regulated or not, according to the activation rule. The spatial triadic network is formed by $N = 10^3$ nodes, with parameter values $c = 0.6$, $c^+ = c^- = 0.2$, $d_r = d_0 = 0.2$, $\rho = 100$, $p = 1.0$.

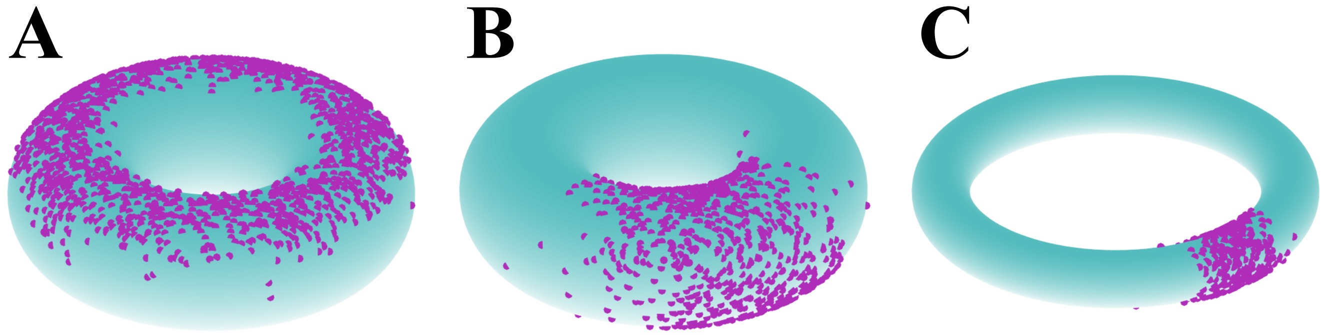

FIG. S-2. Emergent triadic percolation patterns for the square (**A**, **B**) and rectangular (**C**) lattice with periodic boundary conditions (tori). The surface of the torus is shown with turquoise color, and active nodes are shown in pink. In the square torus, the vertical and horizontal dimensions are symmetric, and in the deterministic regime $p = 1$, both horizontal (A) and vertical (B) stripes emerge. In the rectangular torus this symmetry is broken, and in the deterministic regime $p = 1$ stripes emerge only in the shorter direction (e.g. vertical in this exemplary case). The parameters for generating the spatial networks with triadic interactions for panel **A** and **B** are set as $N = 10^4$, $c^+ = c^- = 0.2$, $d_r = d_0 = 0.25$, $c = 0.6$. The size of the square $L = 10$. The parameters for panel **C** are $N = 10^4$, $c^+ = c^- = 0.2$, $d_r = d_0 = 0.25$, $c = 0.4$ and the rectangle has size $L_x = 22.36$, $L_y = 4.47$.

Triadic percolation patterns: self-inhibition and border minimization

In strongly spatial networks (small d_0 and d_r), the emergence of spatial triadic percolation patterns can be understood as a surface minimization effect caused by the underlying local positive and negative regulation mechanisms. Consider the pattern shown in the first panel of Supplementary Fig. $S-1$A (top image), corresponding to a strongly spatial network. Active nodes are shown in pink. In the next step, active nodes will effect their regulation, resulting in structural links being either excited (positive regulation and no negative regulation), inhibited (negative regulation) or not regulated (no active regulatory links). The result of the regulation is shown in the bottom image of the panel, where pink, black and cyan points respectively show excited, inhibited and not-regulated links. The number of active positive and negative regulations of each link depends on its distance to the active regions. Thus, structural links within the active manifold typically have at least one active negative regulation and are inhibited (black points in the bottom panel of Supplementary Fig. $S-1$A). On the other hand, links far from the active regions have no active regulations and thus are not regulated (cyan points). Only at the border between the active and inactive node regions there can be sparse regulation, resulting in a set of links with only positive regulation, shown by the pink points (and another with negative regulation). Consequently, the subsequent pattern (step $t + 1$) roughly corresponds with the border of the current pattern (step t). In this manner, the regulation acts as an effective surface tension, where the tension from inside of the active manifold is caused by negative regulation resulting in self-inhibition, and the tension from outside by the lack of positive regulation.

Surface minimization explains the different observed behaviors. To quantify the temporal aspect, we have defined the overlap between patterns at different time-steps, namely

$$O_\tau(t) = \frac{\sum_i s_i(t)s_i(t+\tau)}{\sum_i s_i(t)}, \tag{S-1}$$

and between the active and inactive patterns at different time-steps, i.e.

$$\bar{O}_\tau(t) = \frac{\sum_i s_i(t)\left(1 - s_i(t+\tau)\right)}{\sum_i s_i(t)}. \tag{S-2}$$

Supplementary Fig. $S-3$ shows O_1, O_2 and $\bar{O}_1$ for $p = 0.1, \ \ldots, \ 1.0$ for the same simulations are reported in Fig. 6 of the main text. For each pattern class, we observe the following dynamics:

1. **Octopus patterns**. The active manifold and its neighbourhood conform the entire network, resulting in *ST-blinking*.

   (a) The overlap between consecutive active states O_1 is close to 0 (dark blue line in Supplementary Fig. $S-3$ for e.g. $p = 0.7$), whereas O_2 is almost one (turquoise line) indicating an approximate period 2 oscillation.

(b) $\bar{O}_1$ is large but smaller than 1 ($0.5 < \bar{O}_1 < 1.0$), indicating that pattern $t+1$ is typically the opposite of pattern t, but fluctuations occur.

(c) If pattern t presents large loops, pattern $t+1$ can present two spatially separated active regions.

(d) The patterns can become unstable due to quenched disorder or random damage (for $p < 1$), causing either continuous deformations (Supplementary Fig. $S-1$A) or the break-down of part of the pattern (Supplementary Fig. $S-1$B).

2. **Small clusters** *diffuse* to nearby positions, and ST-blinking is not observed ($O_2 \approx 0$).

3. **Stripes** emerge for large enough p as they minimize the size of the border (Supplementary Fig. $S-1$B).

(a) Stripes can either *ST-blink* or *diffuse*, see large fluctuations in O_2 for $p > 0.7$ in Supplementary Fig. $S-3$. ST-blinking is characterized by large O_2, and diffusion by small O_2. Note that the typical width of stripes is only one order of magnitude smaller than the size of the system (approx. $1/L$), and consequently effective diffusion is only possible on small time-scales before the stripe returns to the original region.

(b) Multiple stripes can emerge from single stripes if the two neighbourhoods are connected by active structural links (Supplementary Fig. $S-1$B). They require the activation of some longer distance structural links, and therefore are less stable and occur less often than single stripes for typical parameter values. Their emergence is most likely in the case of no random damage ($p = 1$).

Entropy rate

Given a source producing an output that can be described through an ensemble of categorical time-series x, in order to calculate the entropy rate of S, x is divided in non-overlapping motifs or words of length $\hat{L}$. Let $\tilde{p}_i$ be the normalized count of the ith word in the ensemble of words of length $\hat{L}$ in the time-series. Then, the estimate of the entropy rate (in bits per second or steps) is given by

$$H(l) = -\frac{1}{\hat{L}} \sum_i \tilde{p}_i \log_2 \tilde{p}_i. \tag{S-3}$$

The true entropy rate $\hat{H}$ of the source is reached in the limit of infinitely long words, i.e.

$$\hat{H} = \lim_{\hat{L} \to \infty} H(\hat{L}). \tag{S-4}$$

In practice, the entropy rate of the source is estimated by averaging over the entropy rate of an ensemble of individual time-series x, $\hat{H}_x$:

$$\hat{H} = \langle \hat{H}_x \rangle. \tag{S-5}$$

The entropy is a property of sources, and its estimation requires extensive sampling and long time-series that are not always available. Ref. [1] proposed an alternative estimator based on the Lempel-Ziv complexity $LZ76$ [2]. The $LZ76$ is defined recursively. Given the sequence $x_1^n := x_1 x_2, ..., x_n$ of length n ($1 \leq i \leq n$), a block of length n', $B_{n'}$, is a segment of length n' of x_1^n, i.e. $x_{i+1}^{i+n'} := x_{i+1} x_{i+2} ... x_{i+n'}$. We set $B_1 = x_1^1 = x_1$. Suppose that

$$x_1^{n_k} = B_1 B_2, ..., B_k. \tag{S-6}$$

Then, we define

$$B_{k+1} := x_{n_k+1}^{n_{k+1}} \left(n_k + 1 \leq n_{k+1} \leq n\right), \tag{S-7}$$

to be the block of minimal length that does not occur in the sequence $x_1^{n_{k+1}-1}$. By iterating this procedure, we decompose x_1^n in minimal blocks,

$$x_1^n = B_1 B_2, ..., B_p. \tag{S-8}$$

in which all blocks are unique except for (potentially) the last block B_p. The $LZ76$ complexity of x_1^n is defined as the number of blocks in the decomposition (which is unique):

$$C(x_1^n) := p. \tag{S-9}$$

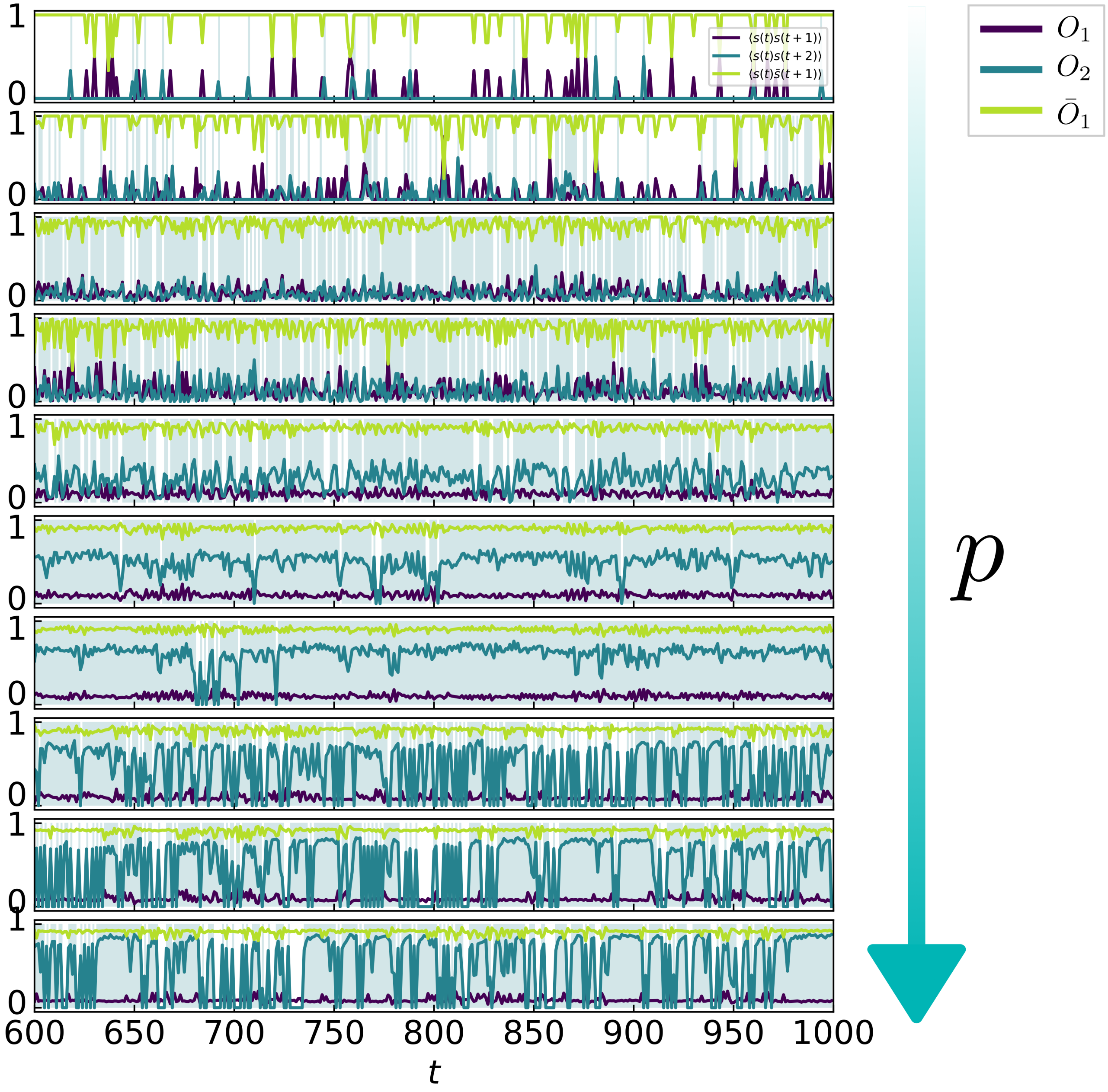

FIG. S-3. Temporal description: Overlap of the triadic percolation patterns. To illustrate the surface tension mechanism, we show the overlap parameters $O_1(t)$ (dark blue), $O_2(t)$ (turquoise) and $\bar{O}_1(t)$ (light green), for different values of $p = 0.1,\ 0.2,\ ...,\ 1.0$, from top to bottom. Shaded areas indicate ST-blinking, i.e. $O_2(t) > \alpha$. These results are for the same network and realizations of the triadic percolation dynamics as those in Fig. 6 of the main text, namely $N = 10^4$, $c^+ = c^- = 0.2$, $d_r = d_0 = 0.25$, $c = 0.4$, $\rho = 100$.

The rate of generation of new patterns along x_1^n is measured by the normalized $LZ76$ complexity

$$c(x_1^n) = \frac{C(x_1^n)}{n/\log_2 n} = \frac{p}{n}\log_2 n. \tag{S-10}$$

Sequences that are not complex have a very small normalized $LZ76$ complexity, and random sequences have maximal $LZ76$ complexity. If a source is stationary, then [1, 3]

$$\limsup_{n\to\infty} c(x_1^n) \le H, \tag{S-11}$$

on average and, if the source is ergodic, then

$$\limsup_{n\to\infty} c(x_1^n) = H, \tag{S-12}$$

almost surely. These equations provide estimates from below to the entropy of a source through the $LZ76$ complexity of the generated time-series. Note that whereas the limit to infinity in Eq. $(S-4)$ is on the word length, which inevitably leads to under-sampling of long words and requires extrapolating the results of short words, the limit in Eq. $(S-12)$ is on the length of the time-series and, if the converge of Eq. $(S-12)$, a reliable estimate can be achieved even in short time-series.

In Supplementary Fig. $S-4$ we show the two procedures described above to estimate the entropy rate, for a set of values of p. Namely, in panel A we show the entropy rate as derived from the normalized $LZ76$ complexity $c(x_1^n)$ when considering an increasing length n of the time-series. As it can be shown, convergence is reached for most values of p. The entropy rate is then measured in this case as $c(x_1^{n=T})$, where T is the length of the time-series. In panel B we show the entropy rate $H(\hat{L})$ of words of increasing length $\hat{L}$ as estimated via Eq. $(S-4)$. The true entropy rate is measured in this case by performing a linear fit to the last two points of the plot (corresponding to $\hat{L} = 1,\ 2$) and extrapolating the trend to $1/\hat{L} = 0$. The results of both estimates are shown in panel C for the same data shown in panels A and B, and in panel D for an ensemble average over 10 iterations of the triadic percolation system and dynamics. As it can be seen, both methods lead to the same results for the cluster-dominated regime (up to the first maximum), but there is a significant deviation between them for the octopus-stripe co-existence region (region around the second maximum). According to Eqs. $(S-11)$ and $(S-12)$, this result points towards the non-ergodicity of the pattern time-series, although more detailed analyses would be required to validate this finding.

Rectangular torus

We consider here the effect of breaking the horizontal-vertical symmetry in the emerging spatial triadic percolation patterns. In order to do so, we simulate the triadic percolation model on a rectangle (width five times its height) with periodic boundary conditions (rectangular torus), whilst all other network and model parameters (in particular the number of nodes, density and scaling of connections with the distance) are kept the same. As shown in Supplementary Fig. $S-2$, stripes always emerge along the shorter dimension in the rectangular torus (see panel C). This is in agreement with the proposed mechanism for the emergence of macroscopic activity patterns, based on the minimization of surface tension. Moreover, we have found that stripes emerge for higher noise values (smaller p) in the case of the rectangular torus, indicating that shorter stripes are more stable.

Barycenter dynamics

For stripe patterns the barycenter in the perpendicular dimension is well-defined. To determine whether stripes predominantly ST-blink or diffuse, we have analyzed the evolution of the barycenter. As a reference, we include also the study of the barycenter for clusters, which can also be defined, and in which case the overlap analysis indicates a diffusive behavior. We consider the case of horizontal stripes and thus focus on the vertical component of the barycenter location.

In Supplementary Fig. $S-5$A and D we show a recurrence plot [5, 6] of the barycenter location along the vertical axis, $y_B(t)$, respectively for small clusters and stripes. We observe that for small clusters the barycenter moves in time through the network, and all locations are reached with homogeneous probability. All points in the recurrence plot fall along the diagonal, indicating a short displacement in each step, due to the small size of the patterns. The situation for stripes is vastly different, and only some locations are visited by the pattern. Moreover, there is a strong preference for a handful of locations. At the microscopic scale, however, deviations of the barycenter position within each macroscopic preferred location are observed over time, indicating that the actual stripe changes over time (in agreement with Fig. 4D in the main text). Points in the recurrence plot fall further from the diagonal in the case of stripes, as these are wider than clusters and the patterns move further on each step. Moreover, the displacements of stripes typically take place in discrete amounts, i.e. stripes typically move the width of one, and occasionally two (in the case of multiple stripes), stripes.

To discriminate whether there is asymmetry in the movement of the patterns, in Supplementary Fig. $S-5$ we display the asymmetry in the movement for each location y, derived from an analysis of the barycenter recurrence plot [5, 6]. In order to do so, we have measured the number of times that the pattern moves up (N_U) and down (N_D) from

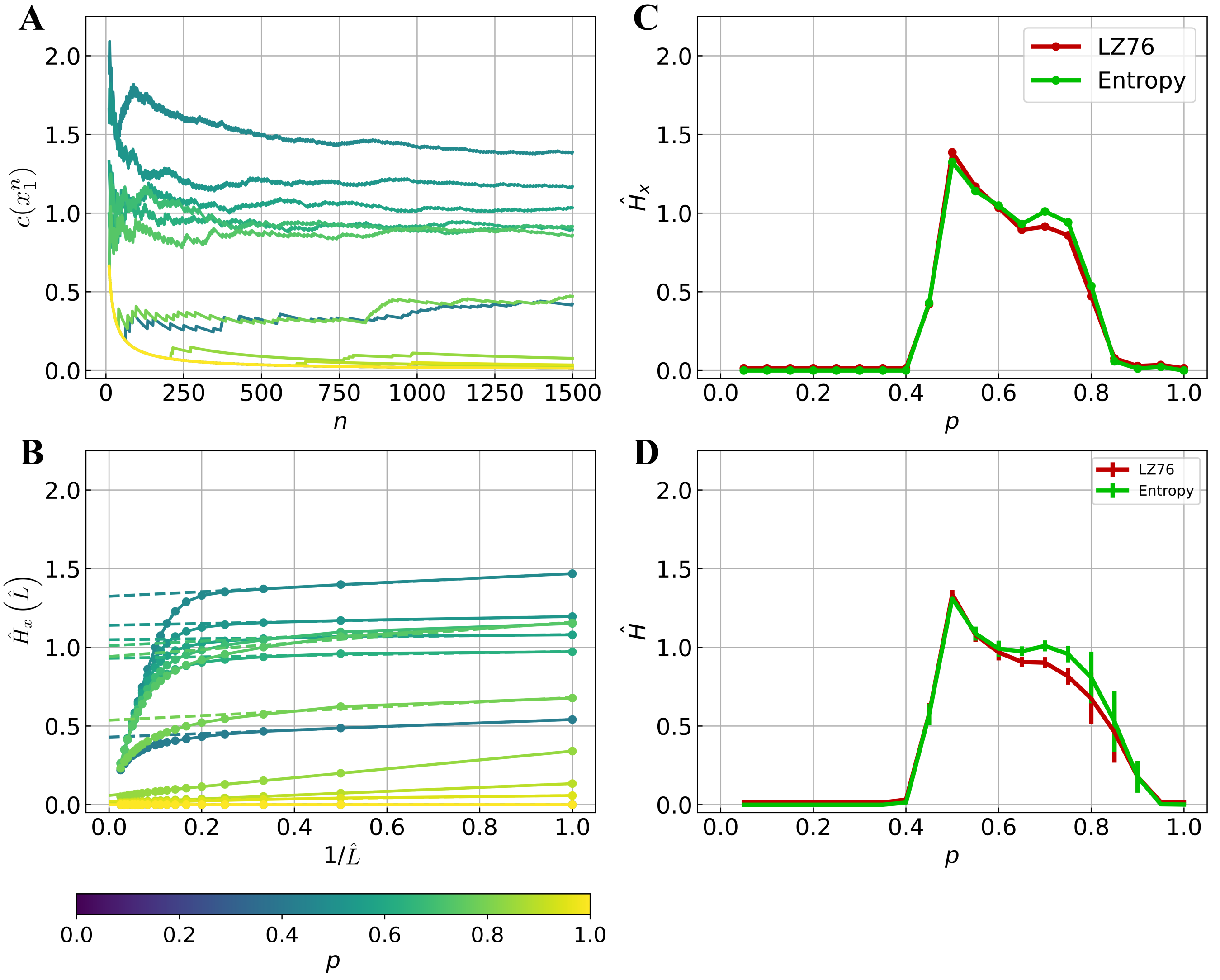

FIG. S-4. Entropy rate estimation. Two procedures were considered to estimate the entropy rate, following Ref. [1]. **A** Normalized Lempel-Ziv 76 complexity $LZ76$ [2, 4], $c(x_1^n)$, of the pattern time-series x for increasing length n. Each line corresponds to a single time-series generated over the same system with different probability of random damage p as indicated by the colorbar. The limit $n \to \infty$ gives the entropy rate, which is estimated here as $c(x_1^{n=T})$ [1], where $T = 1500$ is the integration time. **B** Entropy rate $\hat{H}_x^{\hat{L}}$ of the pattern time-series x when considering motifs or words of increasing length $\hat{L}$. To estimate the entropy rate as $\hat{L} \to \infty$, the linear trend on the right-hand side of the plot is extrapolated to $1/\hat{L} \to 0$ [1]. The data in panel B was generated over the same set of time-series as in panel A. **C** The two estimates of the entropy rate $c(x_1^{n=T})$ and $\hat{H}_x^{\hat{L}\to\infty}$ are shown as a function of the random damage probability p, as indicated by the legend, for the data shown in panels A and B. **D** Ensemble-average of the two estimates of $\hat{H}$, averaged over 10 realizations of the triadic percolation system and dynamics for each value of p. The errorbars indicate the standard deviation. In all panels, the spatial network with triadic interactions is formed by $N = 10^4$ nodes and generated with parameter values $c^+ = c^- = 0.2$, $d_r = d_0 = 0.25$, $c = 0.4$, and we considered a simulation length of 2000 steps. The first 500 steps were discarded as transient.

each location, and report this by the blue and red curves, respectively. Then, the asymmetry at each location $A(y)$ is measured as the difference between N_U and N_D (black curve in the bottom panels). To measure $A(y)$ we made use of a coarse-grained grid of size 30×30, equal to the one used for the spatial characterization of the patterns in the main text. No significant asymmetry is found for clusters, whereas for stripes there are two distinct locations, with positive and negative asymmetry respectively, such that the stripe ST-blinks between them (i.e. there is a large probability of moving upwards from the lower location, and conversely of moving downwards from the upper position). This asymmetry arises due to quenched disorder in the structural and regulatory networks, and leads to ST-blinking being the preferred mechanism for $p = 1$. We note that this dynamics cannot be observed clearly through $R(t)$, or through

$H(t)$ or $C(t)$, as stripes are typically of the same size and shape.

Finally, we have measured the return probability $P_0(\tau)$ of the barycenter to a given location after τ steps (see Supplementary Fig. $S-5$C, F respectively for clusters and stripes). A return event after τ steps occurs if $x_B(t)$ and $x_B(t+\tau)$ belong to the same cell of the coarse-grained 30×30 grid. $P_0(\tau)$ confirms that clusters show a power-law decay of $P_0(\tau)$ on short to intermediate time-scales, until a plateau is reached. On the contrary, the decay for stripes is slower and a plateau is reached earlier. We also notice a significant difference for odd and even times for stripes due to the self-inhibition effect.

In summary, diffusion only emerges at short time-scales, due to quenched disorder caused by local inhomogeneities. In general, the probabilities of moving upwards and downwards (or right and left in the case of vertical stripes) are not equal and depend on the position, effectively trapping the stripe into preferred locations with only short visits to other network regions, resulting in an inhomogeneous spatial distribution of activity over time.

Spatial networks used to generate Supplementary Movies

We further provide movies of spatio-temporal patterns in the Supplementary Information. Four Supplementary movies show the ST-blinking of stripes, ST-blinking of octopus, the intermittency between stripes and octopus, and sustaining stripes. We list the parameters for generating these movies as follows.

- ST-blinking of strips (Video 1): The parameters for generating the movie are $c^+ = 0.2$, $c^- = 0.2$, $p = 1.0$, $c = 0.4$, $d_r = d_0 = 0.25$. Patterns of 50 time steps are shown.

- ST-blinking of octopus (Video 2): The parameters for generating the movie are $c^+ = 0.2$, $c^- = 0.2$, $p = 1.0$, $c = 0.4$, $d_r = d_0 = 0.25$. Patterns of 50 time steps are shown.

- Intermittency between stripes and octopus (Video 3): The parameters for generating the movie are $c^+ = 0.2$, $c^- = 0.2$, $p = 1.0$, $c = 0.4$, $d_r = d_0 = 0.25$. Patterns of 50 time steps are shown.

- Sustaining stripes (Video 4): A period-6 oscillation is shown. The parameters for generating the movie is $c^+ = 0.4$, $c^- = 0.2$, $p = 1.0$, $c = 0.4$, $d_r = d_0 = 0.25$. Patterns of 50 time steps are shown.

Extended parameter space

While in the main body of this work we discuss the dependence of the triadic percolation patterns as a function of p, in this section we investigate the dependence of the spatial patterns in an extended parameter space by varying other parameters of the model.

Dependence on $d_r = d_r^+ = d_r^-$ - The parameter d_r characterizes the scaling of the regulatory links with the distance. For small d_r regulatory links are local and span short distances, whereas for large d_r regulatory links are long and may span the whole network. Here we consider the effect of d_r of the emergent spatio-temporal dynamics, fixing the remaining parameters as in the main text ($N = 10^4$, $\rho = 100$, $d_0 = 0.25$, $c = 0.4$, $c^\pm = 0.2$). We make two remarks: i) by changing d_r the number of regulatory links also changes, and ii) the parameter controlling the length of the structural links, d_0, remains fixed, and thus structural links remain spatially organized. Finally, to remove the effect of annealed disorder on the dynamic caused by the random link deactivation, we focus on the deterministic case with $p = 1$.

The results of this analysis are illustrated in Figure S-6. We found that, for intermediate d_r, the system displays chaotic dynamics involving stripes (S) and octopus (O) patterns, depending on the particular realization of the quenched disorder. For very small d_r the network becomes disconnected due to the lack of positive regulations, and the system is in a stationary state. A small increase in d_r, with $d_r < d_0$, leads to high-activity homogeneous states, with $R \to 1$ and no spatial organization. In this regime, each structural link is regulated by a handful of nodes (in the example shown in Figure S-6 each structural link has on average 0.37 positive and 0.37 negative regulators), but the structural link density is relatively high (in the example shown, the mean degree is 15.78). Thus, most nodes have at least one link that is positively but not negatively regulated (9607 in this example), and a high-density state emerges as a stable state. Depending on the particular quenched noise realization, periodic oscillations in which the state of a few nodes ($n \ll N$) changes may arise instead.

If $d_r > d_0$, periodic oscillations emerge, which may involve cluster and octopus states, cluster-anticluster transitions, and also the disconnected state. Here by anticluster we refer to the highly active state with only a small cluster of

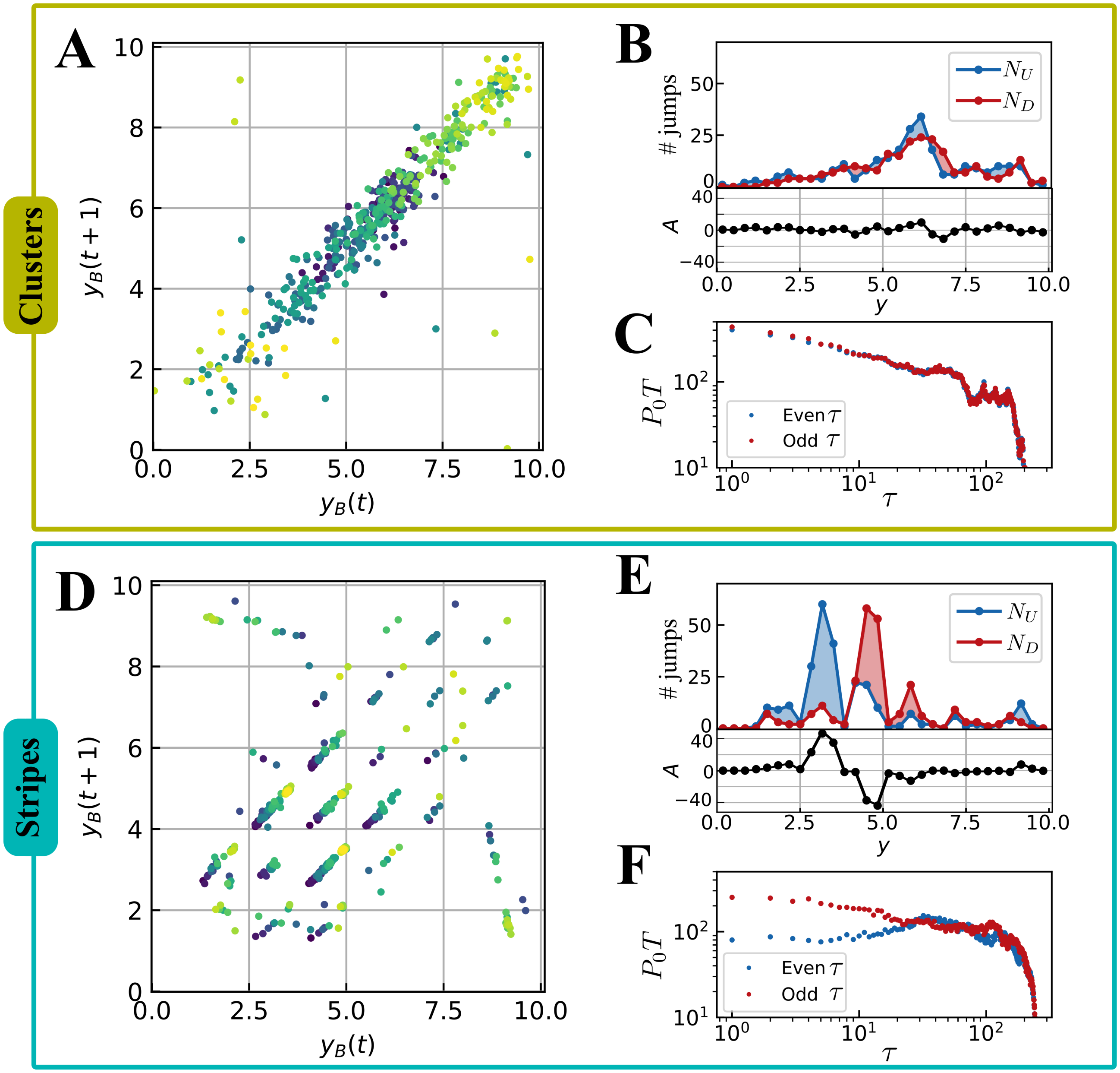

FIG. S-5. Barycenter dynamics of the spatial triadic percolation patterns along the vertical axis corresponding to small clusters ($p = 0.3$, panels A-D) and horizontal stripes ($p = 1.0$, panels E-H). **A**, **D** Recurrence plot of the barycenter location y_B with lag $\tau = 1$. Each point corresponds to a time-step, with the time indicated by the color scale (from dark blue for $t = 600$ to yellow for $t = 1000$). **B**, **E** Asymmetry in the movement of the barycenter. The top plot shows the number of times the barycenter moves up (N_U, blue points) and down (N_D, red points) from each position. The bottom plot shows the asymmetry at each position, $A(y)$, defined as $A(y) = N_U(y) - N_D(y)$. **C**, **F** Number of return events $P_0(\tau)T$ (i.e. the return probability $P_0(\tau)$ times the total number of steps considered T). Odd and even times have been separated as they follow different scaling for stripes due to the self-inhibition effect. These results are for the same network and realizations of the triadic percolation dynamics as those in Fig. 6 of the main text, namely $N = 10^4$, $c^+ = c^- = 0.2$, $d_r = d_0 = 0.25$, $c = 0.4$, $\rho = 100$, $T = 400$.

inactive nodes. (We distinguish the anticluster from the octopus state as the anticluster loses the tubular organization of octopus and stripe patterns.) Finally, for large d_r, the spatial organization of the regulatory links disappears, and a blinking dynamic emerges between a low activity state and an intermediate activity state.

Dependence on d_r^+ and d_r^- - We have considered the effect of different spatial distributions of the positive (d_r^+) and negative (d_r^-) regulations by sampling the (d_r^+, d_r^-) space. The numerical results confirm that spatial patterns and

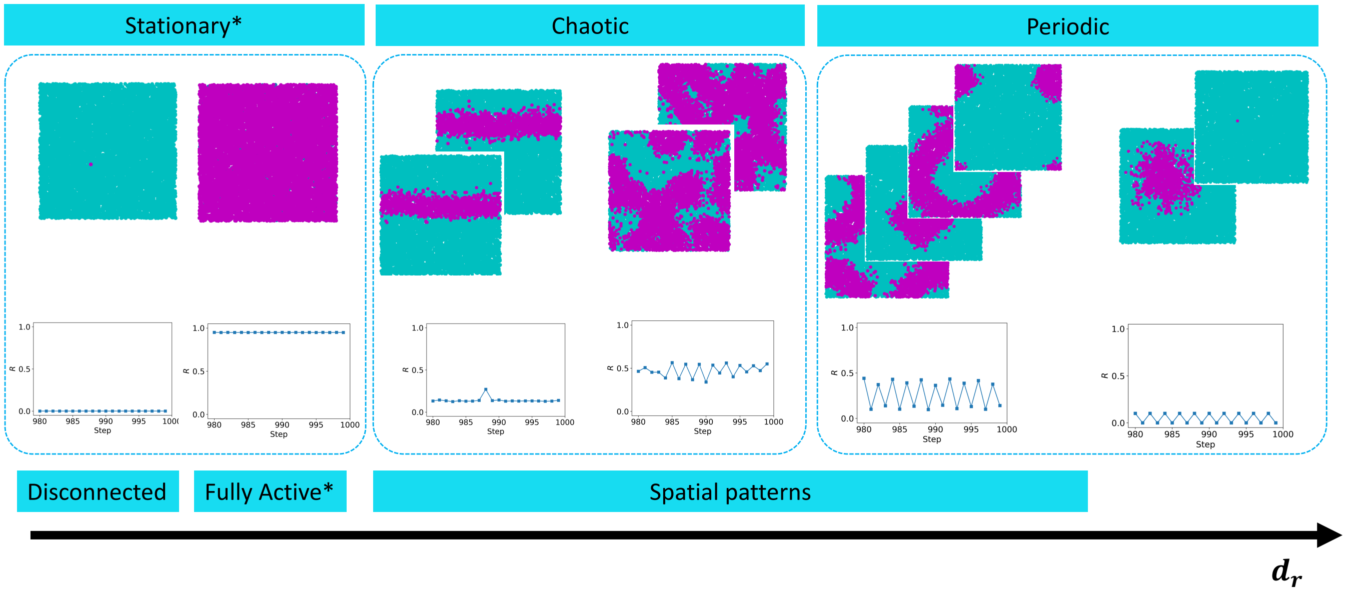

FIG. S-6. Effect of d_r on the deterministic ($p = 1$) triadic percolation dynamics. Parameters are $N = 10^4$, $c = 0.4$, $c^+ = c^- = 0.2$, $\rho = 100$, $d_0 = 0.25$, $T = 10^3$. From left to right, $d_r = 0.01,\ 0.05,\ 0.25$. In each panel, the time series of network activity $R(t)$ and the corresponding patterns are shown. In the second and the third panels, the time evolution of patterns is shown by the overlapping patterns.

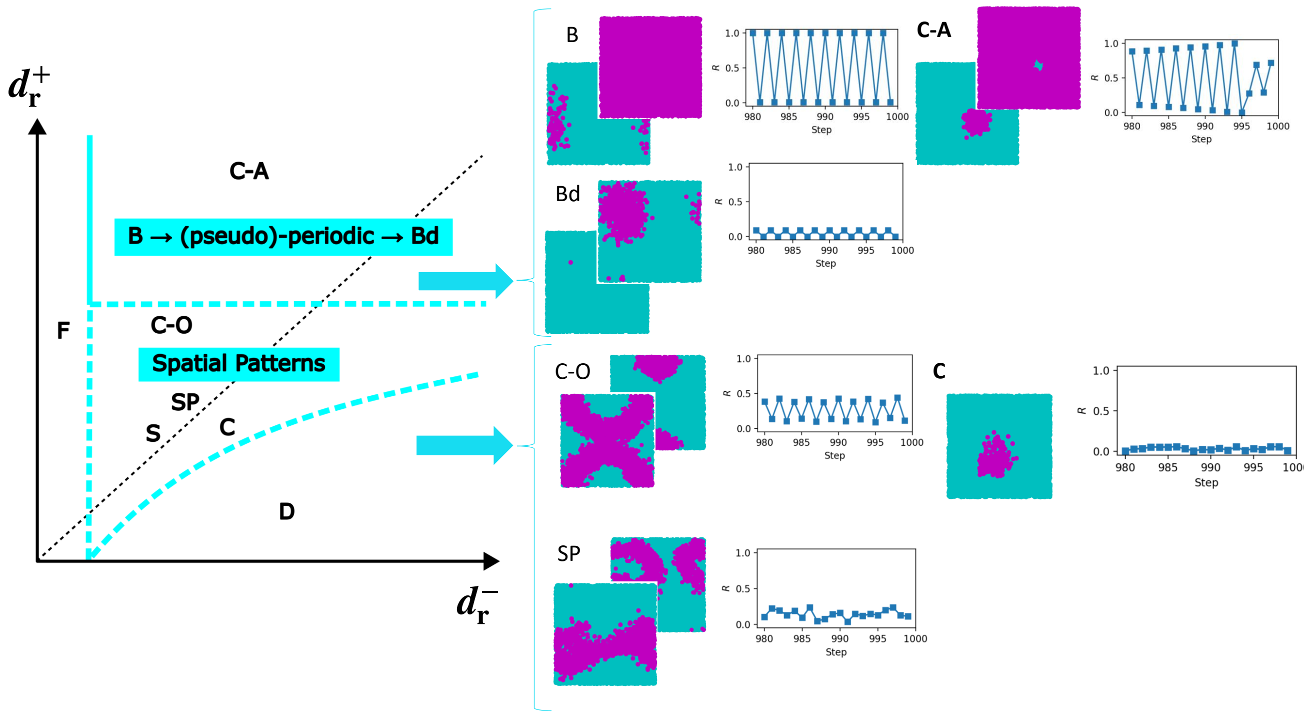

FIG. S-7. Effect of $d_r^{\pm}$ on the deterministic ($p = 1$) triadic percolation dynamics. Parameters are $N = 10^4$, $c = 0.4$, $c^+ = c^- = 0.2$, $\rho = 100$, $p = 1.0$, $T = 10^3$. The diagram summarizes the different regimes that emerge in the (d_r^+, d_r^-) parameter space in a conceptual manner. The location of each regime and the transition lines are approximate. The regimes are: (near) fully active, $R \approx 1$ (F), disconnected (D), blinking (B), periodic (or pseudo-periodic) oscillations, including cluster-anticluster (C-A), cluster-octopus oscillations, small clusters (C) and spatial patterns including stripes (S) and more general time-series involving stripes, octopus and clusters (SP).

complex temporal dynamics emerge for intermediate values of $d_r^\pm$ and in particular close to the diagonal ($d_r^+ \approx d_r^-$). In the outer sections of the (d_r^+, d_r^-) we recover the trivial cases found in the previous section. In particular, for large $d_r^\pm$ the regulations are no longer spatial, as discussed in the previous section, and blinking between the cluster and disconnected state (Bd) emerges. Note that the actual scale for the regimes described below depends on the system parameters, in particular the density of nodes (ρ) and structural links (c) and the spatial scaling of the structural links (d_0). For small $d_r^- \ll 1$ there are few (and local) negative regulations, and the near fully active state is stable (F) As d_r^- increases the giant component becomes less dense and generally remains homogeneously distributed. On the other hand, if negative regulations dominate ($d_r^- \gg d_r^+$), the disconnected state (D) is stable. Remarkably, the type of transition from the highly active homogeneous regime when d_r^- increases depends on d_r^+. For spatially organized positive regulations (d_r^+ small), the giant component gets gradually smaller until cluster states emerge (C). In this regime and around the balanced case ($d_r^+ = d_r^-$) spatial patterns (SP), including octopus (O), stripes (S) and clusters, with complex spatio-temporal dynamics emerge. Depending on whether there is an offset of positive or negative regulations, cluster-octopus oscillations (dominant positive regulations) or cluster states (dominant negative regulations) emerge. On the contrary, for large d_r^+, the network blinks between a high activity ($R \to 1$) and a low activity ($0 < R \ll 1$) states (B). Further increasing d_r^- leads to periodic oscillations of different periods (see the 5th case in Figure S-6) and finally to blinking between the disconnected and cluster states (Bd). For large d_r^+ and intermediate d_r^- cluster-anticluster oscillations (C-A), sometimes through the disconnected state (i.e. cluster to anticluster to disconnected state) are the most common behavior.

Dependence on the ratio c^+/c^-- We have also investigated the emergent behavior of the system when the ratio c^+/c^- is changed (data not shown). To illustrate this dependence, here we describe the dependence of the dynamical patterns on the ratio c^+/c^- observed for parameters $d_0 = d_r = 0.25$, $c = 0.6$, $p = 1.0$, $c^- = 0.2$ as c^+ varies from 0.1 to 1. The general trend described hereby is robust for a wide range of parameters p and c.

For c^+/c^- small only small size activation clusters appear. For intermediate values of c^+/c^- a metastable phase with alternating activation patterns emerges, including periodic, chaotic and single and multiple stripe-like patterns. Finally, for large c^+/c^- only single stripe-like blinking patterns occurs in the system dynamical behavior.

[1] José M Amigó, Janusz Szczepański, Elek Wajnryb, and Maria V Sanchez-Vives, "Estimating the entropy rate of spike trains via lempel-ziv complexity," Neural Computation **16**, 717–736 (2004).

[2] Abraham Lempel and Jacob Ziv, "On the complexity of finite sequences," IEEE Transactions on information theory **22**, 75–81 (1976).

[3] Jacob Ziv, "Coding theorems for individual sequences," IEEE Transactions on information theory **24**, 405–412 (1978).

[4] F Kaspar and HG Schuster, "Easily calculable measure for the complexity of spatiotemporal patterns," Physical review A **36**, 842 (1987).

[5] Jean-Pierre Eckmann, S Oliffson Kamphorst, David Ruelle, *et al.*, "Recurrence plots of dynamical systems," World Scientific Series on Nonlinear Science Series A **16**, 441–446 (1995).

[6] Norbert Marwan, M Carmen Romano, Marco Thiel, and Jürgen Kurths, "Recurrence plots for the analysis of complex systems," Physics reports **438**, 237–329 (2007).
