# Supplementary figures and images for: Triadic percolation induces dynamical topological patterns in higher-order networks
Source: PNAS Nexus. 2024 Jul 9;3(7):pgae270. doi: 10.1093/pnasnexus/pgae270 (PMC11259606; doi:10.1093/pnasnexus/pgae270)

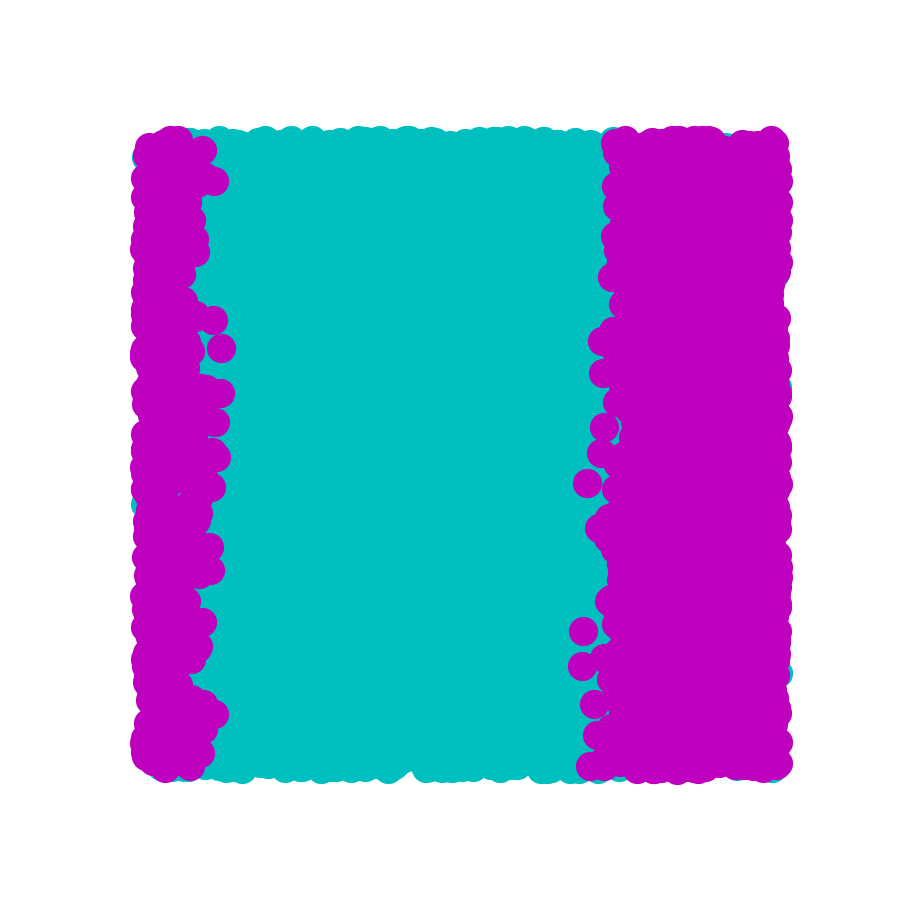

Supplement: pgae270_Supplementary_Data [file pgae270_supplementary_data.zip › PNASNEXUS-PNASNEXUS-2024-00029R-s01.gif]

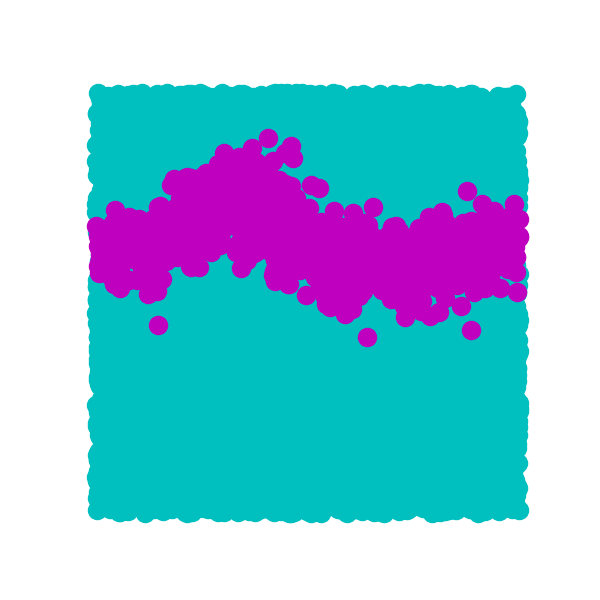

Supplement: pgae270_Supplementary_Data [file pgae270_supplementary_data.zip › PNASNEXUS-PNASNEXUS-2024-00029R-s02.gif]

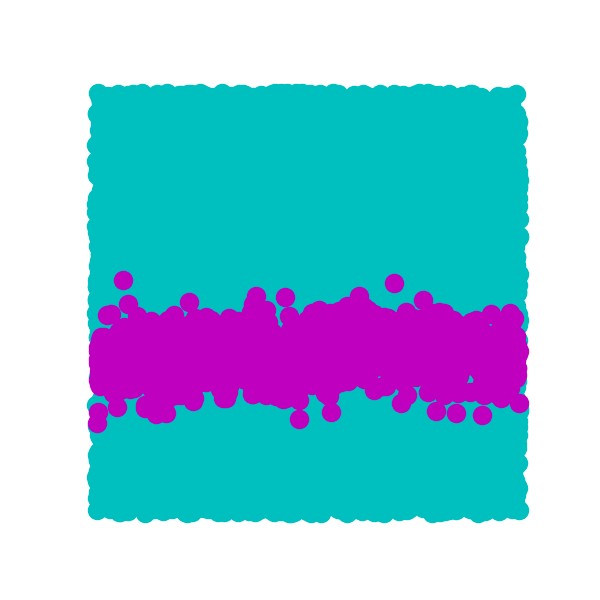

Supplement: pgae270_Supplementary_Data [file pgae270_supplementary_data.zip › PNASNEXUS-PNASNEXUS-2024-00029R-s03.gif]

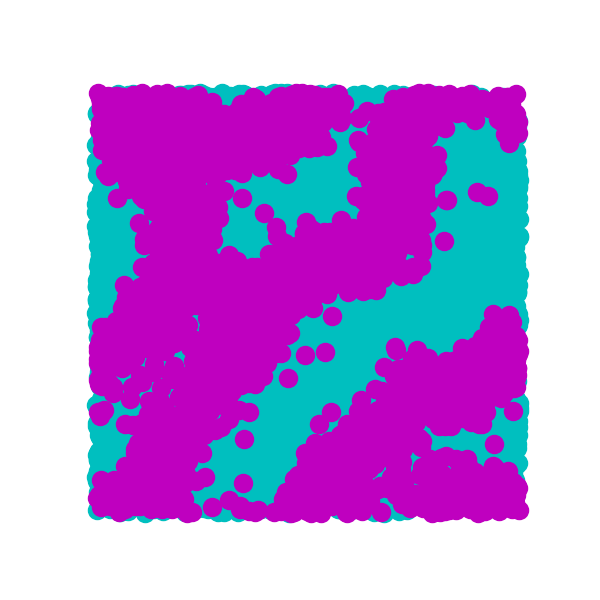

Supplement: pgae270_Supplementary_Data [file pgae270_supplementary_data.zip › PNASNEXUS-PNASNEXUS-2024-00029R-s04.gif]
